# Stochasticity of poly(2-oxazoline) oligomer hydrolysis determined by tandem mass spectrometry†

**DOI:** 10.1039/d2py00437b

**Published:** 2022-06-02

**Authors:** Tomos E. Morgan, Thomas G. Floyd, Bryan P. Marzullo, Christopher A. Wootton, Mark P. Barrow, Anthony W. T. Bristow, Sébastien Perrier, Peter B. O'Connor

**Affiliations:** Department of Chemistry, University of Warwick Coventry CV4 7AL UK p.oconnor@warwick.ac.uk; Chemical Development, Pharmaceutical Technology and Development, Operations, AstraZeneca Charter Way Macclesfield SK102NA UK; Warwick Medical School, University of Warwick Coventry CV4 7AL UK; Faculty of Pharmaceutical Sciences, Monash University 381 Royal Parade Parkville VIC 3052 Australia

## Abstract

Understanding modification of synthetic polymer structures is necessary for their accurate synthesis and potential applications. In this contribution, a series of partially hydrolyzed poly(2-oxazoline) species were produced forming poly[(2-polyoxazoline)-*co*-(ethylenimine)] (P(EtOx-*co*-EI)) copolymers; EI being the hydrolyzed product of Ox. Bulk mass spectrometry (MS) measurements accurately measured the EI content. Tandem mass spectrometry analysis of the EI content in the copolymer samples determined the distribution of each monomer within the copolymer and corresponded to a theoretically modelled random distribution. The EI distribution across the polymers was shown to be effected by the choice of terminus, with a permanent hydrolysis event observed at an OH terminus. A neighbouring group effect wasn't observed at the polymer length analysed (approximately 25-mer species), suggesting that previously observed neighbouring group effects require a larger polymer chain. Although clearly useful for random polymer distribution this approach may be applied to many systems containing non-specific modifications to determine if they are directed or random locations across peptides, proteins, polymers, and nucleic acids.

## Abstract 2

Tandem mass spectrometry can be used to better understand modification sites of synthetic polymer structures providing more complete chemical knowledge which is necessary for their accurate synthesis and potential applications.

## Graphical abstract

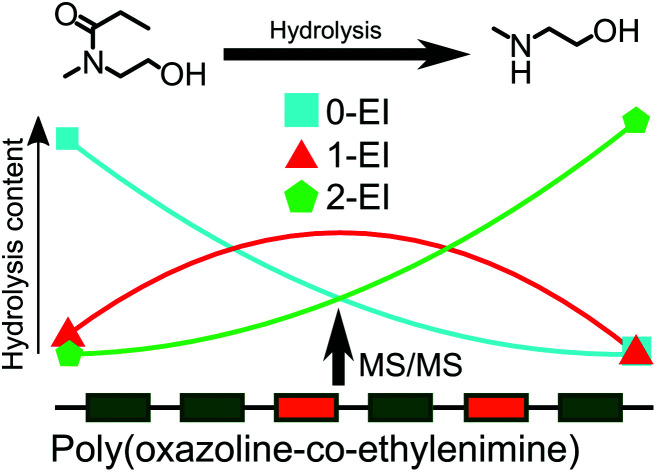

## Introduction

The use of biocompatible poly(2-oxazoline)s (PEtOx) as an alternative to poly(ethylene glycol) (PEG)-based bioconjugation species is well established due to favorable immunogenic properties^1–3^ and ease of introducing functionality.^4,5^ Modification of PEtOxs by partial hydrolysis to poly[(oxazoline)-*co*-(ethylenimine)] P(EtOx-*co*-EI) copolymers offers an advantage as further post polymerization modification can be readily controlled,^6^ biocompatibility compared with polyethylenimine (PEI) homopolymers is increased^7–9^ and biological response can be fine-tuned.^10,11^ As EI is positively charged in biological media, P(EtOx-*co*-EI) can transport poly(nucleotides) as a therapeutic agent, protecting the cargo from degradation and improving the biocompatibility compared to commonly used transfection agents.^9,12–14^ Biological properties of P(EtOx-*co*-EI) are based on the size, initiating and terminating groups, and the levels and distribution of EI present.^15,16^ The precise knowledge of EI groups concentration and location along the chain is key to assess gene delivery properties and toxicity.

Electrospray ionisation (ESI) mass spectrometry (MS) analysis of PEtOx and PEI has been previously studied.^17,18^ Copolymer mass spectrometry can be simplified using Kendrick^19^ mass analysis, separating copolymers by their monomeric content *via* fractional mass calculation.^20–25^ The separation of different fragmentation pathways and monomer unit composition allows the rapid *de novo* elucidation of highly complex tandem MS spectra. Tandem MS (MS/MS) methods discern copolymer sequence,^26–30^ giving insights into reaction mechanism such as reactivity ratios.^31^ Use of collision induced dissociation (CID)^17,32–34^ and electron capture dissociation (ECD)^26,27,35–37^ methods have been used for analysis of PEtOxs and PEIs, giving sequence coverage and initiating and terminating end group information.

The partial hydrolysis of a PEtOx to PEI can be well controlled,^9,38^ nuclear magnetic resonance (NMR) measures the bulk copolymer ratio but not the distribution of hydrolysis or variation with polymer size. Hydrolysis kinetics for PEtOx has been extensively studied,^39^ and the rate of hydrolysis is accelerated at the beginning of the reaction. A neighboring group effect has been postulated, whereby an EI adjacent to an EtOx can activate the carbonyl towards nucleophilic attack *via* an intramolecular hydrogen bond,^9^ theoretically producing pseudo-block structures. Localization of non-specific modifications *via* MS/MS has been effectively carried out using DNA,^40^ we extend this approach by comparing theoretical random hydrolysis distributions along a polymer backbone.

In this study, we report the bulk and sequence characterization of P(EtOx-*co*-EI)-OH and P(EtOx-*co*-EI)-N_3_ copolymers *via* MS. The bulk hydrolysis measurements between MS and NMR closely align and tandem mass spectrometry gave insight into the randomness of distribution of the EI species, previously unexplored by other methods as well as uncovering the effects of the terminal group on the hydrolysis.

## Experimental section

### Synthetic procedures

**Scheme 1:**
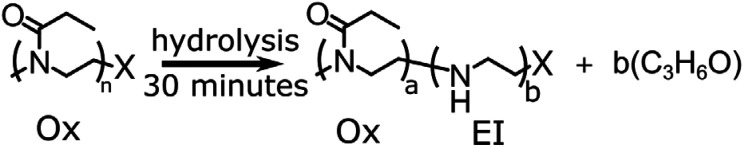
Overview of synthesis of P(EtOx-*co*-EI), through hydrolysis of POx.

Cationic ring opening polymerisation of 2-ethyl-2 oxazoline was initiated by methyl tosylate and terminated by addition of sodium hydroxide (NaOH) or sodium azide (NaN_3_) producing an α-methyl-end group and an ω-hydroxyl- or azide capped poly(2-ethyl-2-oxazoline), Section S1 in ESI.† The resulting poly(2-ethyl-2-oxazoline) was hydrolysed by dissolving the polymer in 1 M HCl, at an amide concentration of 0.48 M and heating to 120 °C for 30 minutes using a microwave reactor yielding the P(EtOx-*co*-EI) copolymer. The total amount of hydrolysis was calculated by NMR using equn (S1) in the ESI.† A full synthetic procedure is given in the ESI Section S2,†Scheme 1 shows an overview of the hydrolysis process.

The hydrolysed sample was dissolved into purified water obtained from a Direct-Q3 Ultrapure Water System (Millipore, Lutterworth, United Kingdom) at 20 μM and acidified for analysis *via* addition of 0.5% formic acid (v/v) (Sigma-Aldrich, Dorset, United Kingdom). All experiments were performed on a 12 T solariX Fourier transform ion cyclotron resonance mass spectrometer (Bruker Daltonik, GmbH, Bremen, Germany) using a nano-electrospray (nESI) ion source in positive-ion mode. The ECD was carried out with the use of an indirectly heated hollow cathode with a current set at 1.5 A, with a pulse length of 0.2 s and bias 1.2 V. All data were recorded using 4 mega-word (2^22^, 22 bit) transients (1.6777 s) achieving approximately 500 000 resolving power at *m*/*z* 400 for the intact mass spectrometry with a mass cut off at *m*/*z* 147 and 400 000 resolving power at *m*/*z* 400 for the tandem mass spectrometry with a low mass cut off at *m*/*z* 100. All mass spectra were internally calibrated by the intact polymer peaks across the polymer distribution, or by internal calibration of fragment peaks in ECD spectra (peaks used for calibration are marked). The peaks used for internal calibration were crosschecked using both the *a* and *x* fragment series. The Bruker SNAP algorithm was used for peak picking with the polyoxazoline monomer used as the repeat unit (C_5_H_9_NO). The Bruker SNAP algorithm matches a calculated isotope distribution adjusted to a repeat unit with increasing mass.^41–43^

**Fig. 1:**
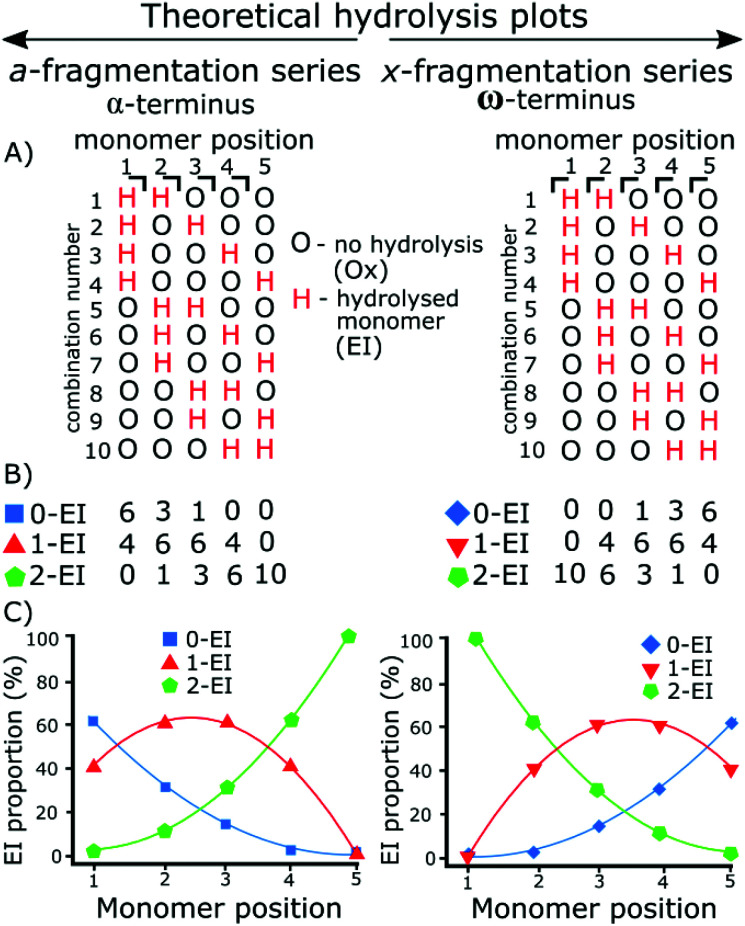
Theoretical plots of all possible combinations of hydrolyzed species (H) and unhydrolyzed species (O) for a 5-monomer polymer where 2 units are randomly hydrolyzed. (A) All 10 combinations of the random distribution of 2 hydrolyzed species in a 5-monomer polymer are represented here, each H represents the presence of 1-EI unit where there are two Hs present the fragment is a 2-EI containing fragment. The *a*- and *x*-series continue to opposing termini meaning the distribution is a mirror image. (C) The distribution plotted as a percentage, as there are ten combinations generated then each proportion from (B) is plotted at each monomer position. Taking monomer position 2 in the *a*-series: there are 3 0-EI fragments, 6 1-EI fragments, and 1 2-EI fragments, plotted below as 30% 0-EI, 60% 1-EI, and 10% 2-EI.

The use of tandem mass spectrometry to localize non-specific modification positions graphically has been effectively carried out using DNA,^40^ we extend this by predicting and then fitting to, random distributions. The fragmentation data was compared to the statistically distributed fragmentation patterns. The statistically distributed hydrolysis maps were calculated by combination of PEI units within a polymer chain using a modified Heap's algorithm.^44^ The total number of arrangements was calculated and the fragment intensities were calculated by code included in the ESI.†Fig. 1 shows a theoretical model of 2 EI units evenly distributed across five monomer units using the Heap's algorithm and how, at different fragmentation points, the total proportion of each species will vary. Put simply:

Random hydrolysis events (H) will evenly distribute across all possible combinations. All possible combinations will be statistically represented during the analysis. At monomer position 1 measuring back to the α (left) methyl terminus 40% of fragments have one hydrolysis event (H) as only one monomer unit is present; a doubly hydrolysed species can't be present. The remaining 60% of fragments possible have not undergone a hydrolysis event. One hydrolysis event (H) represents the presence of and EI species. Depending on whether the fragment contains 0, 1, or 2 hydrolysis events (H) dictate whether that fragment is a 0-EI, 1-EI, or 2-EI containing species respectively.

Moving to monomer position 2 60% of measured fragment oligomers contain one hydrolysis event (H). 30% of fragments contain no hydrolysis events and 10% of fragments contained 2 hydrolysis events.

Fragmentation at each monomer and the resulting oligomer unit can be analyzed in the same way and the proportions compared. If the practical data shows similar binomial distribution to the theoretical plot then they hydrolysis is random, if there is a large shift in the distribution then it is not random.

Practically, the peak areas at each monomer position are compared. For example, the 0-EI *a*_3_, 1-EI *a*_3_, and 2-EI *a*_3_ fragment peak areas are compared to one another. The peak area is calculated within the data analysis program and the same peak picking is used for all assignments. As the measurement is relative to other peaks in each summed spectrum, deviations in signal to noise from spectrum to spectrum do not influence the techniques use, and fragments are similar enough in abundance and resolved well enough that S/N variation has little effect on individual monomer positions.

## Results and discussion

### Intact mass spectrometry characterisation

**Fig. 2:**
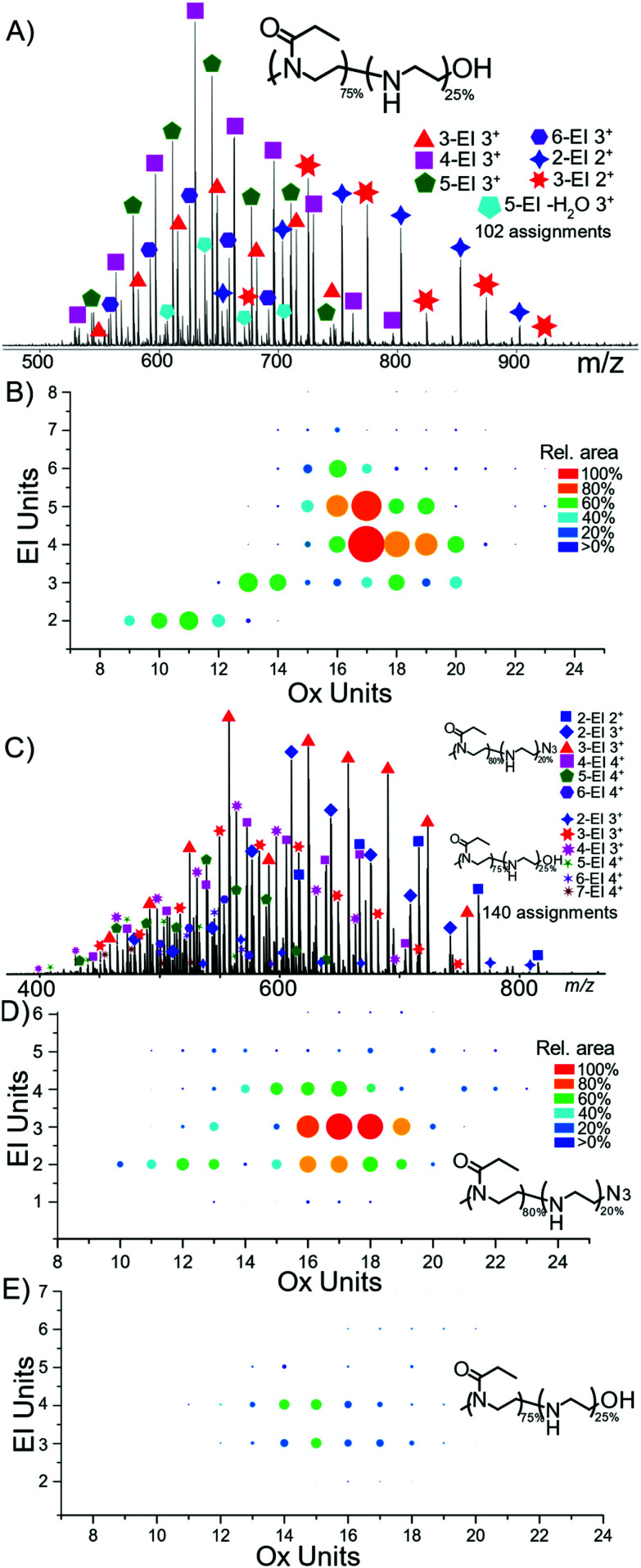
(A) nESI analysis of P(EtOx-*co*-EI)-OH, with (B) heat map of PEtOx units to EI units spot area directly relates to peak area in the MS, (C) nESI analysis of P(EtOx-*co*-EI)-N_3_ (D) and (E) corresponding heat maps to the same scale indicating more N_3_ terminated copolymer was present compared to OH terminated.

Nano-Electrospray ionisation (nESI) produced mainly protonated adduct species between charges 2+ and 4+, Fig. 2. The distribution of hydrolysis based on polymer length shows that the majority of ion signal is concentrated into a small number of species. At shorter polymer lengths the amount of hydrolysis seems to increase proportionally with length shown clearly in Fig. 2B, with a diagonal increase with EtOx length and number of EI units. The percentage values represent the amount of bulk hydrolysis in each polymer species, specified values later in the manuscript represent single isolated species for tandem MS analysis.

The weighted average hydrolysis value closely aligned to the amounts measured by NMR, presented in the SI. Average bulk hydrolysis level of P(EtOx-*co*-EI)-OH was 25% by NMR. By MS the degree of hydrolysis is 25.8% demonstrating parity between MS and NMR measurements, MS also achieves species-specific information in addition to the distribution of the hydrolysis by mass. Over 100 assignments were made for each of the copolymer species, detailed in the ESI.†

The monomer distribution can be plotted *via* heat maps corresponding to the EtOx and EI content (Fig. 2B, D and E). The mass range of P(EtOx-*co*-EI)-OH started with a mass of 1337 Da consisting of 5-EI units and 11-EtOx units to 2568 Da containing 6-EI and 23-EtOx units. The smallest polymer detected was formed of 9-EtOx and 2-EI units.

The sample of P(EtOx-*co*-EI)-N_3_ contained both P(EtOx-*co*-EI)-N_3_ and P(EtOx-*co*-EI)-OH. Both copolymer species could be separated by mass and their individual hydrolysis levels measured. The largest detected P(EtOx-*co*-EI)-N_3_ polymer of 25 EtOx units and 4 EI units and highest mass of 2704 Da. The smallest polymer contained 10 EtOx units and 2 EI units and was also the lowest mass at 1232 Da. The end groups were retained and detected throughout the analysis.

### Statistical modelling of stochasticity

Fig. 2D and E are normalised to the same area and show the difference in abundance of N_3_ terminated and OH terminated polymer present within the N_3_ polymer sample. Overall, this shows that the OH sample is present at, on average, a shorter polymer length but with a higher hydrolysis amount per polymer.

### Tandem mass spectrometry characterisation

Electron capture dissociation (ECD) fragmentation of polyoxazolines fragments the nitrogen–carbon bond adjacent to the side chain. Fragmentation generates *a*-series fragments which contain the α-terminus and *x*-series fragments that contain the ω-terminus.

Tandem mass spectrometry using ECD was carried out on the hydrolysed copolymer species.^45^ The fragmentation data was compared to the statistically distributed fragmentation patterns.

**Fig. 3:**
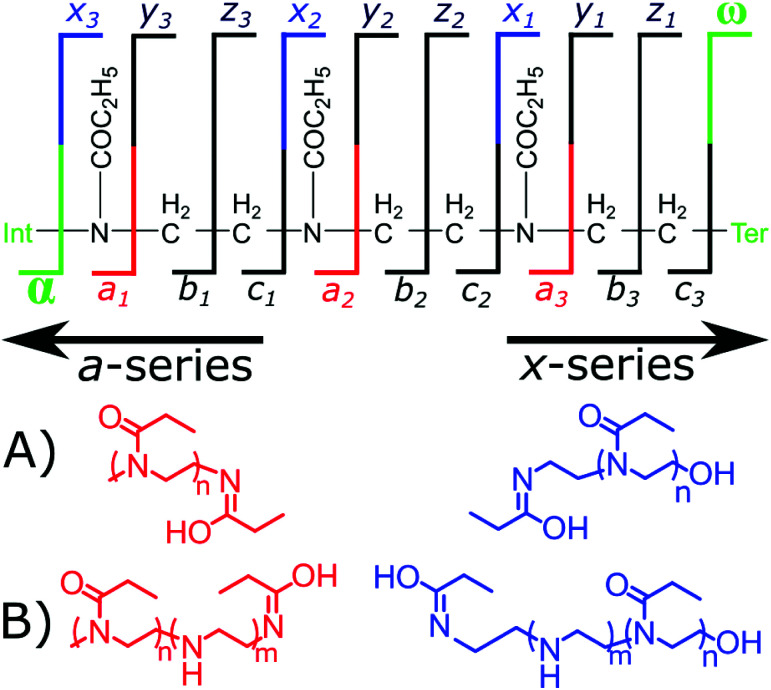
Cleavage diagram of a polyoxazoline polymer with the observed *a*- and *x*-series fragments, (A) the expected *a*-series and *x*-series fragments produced from a homopolymer and (B) the expected *a*-series and *x*-series fragments produced from an EI/EtOx copolymer.

Randomly distributed hydrolysis maps were calculated by combination of EI units within a polymer chain using a modified Heap's algorithm.^44^ The Heap's algorithm generates a plot of the random distribution of multiple hydrolysis events across a polymer backbone, the formula is included in the (ESI†). The fragmentation of the copolymers by ECD gave two fragment ion series *a* and *x*, Fig. 3. The *a* fragmentation series contains the α terminus, which is a methyl group in both polymers studied herein. The *x* series contains the ω terminus, OH or N_3_.

**Fig. 4:**
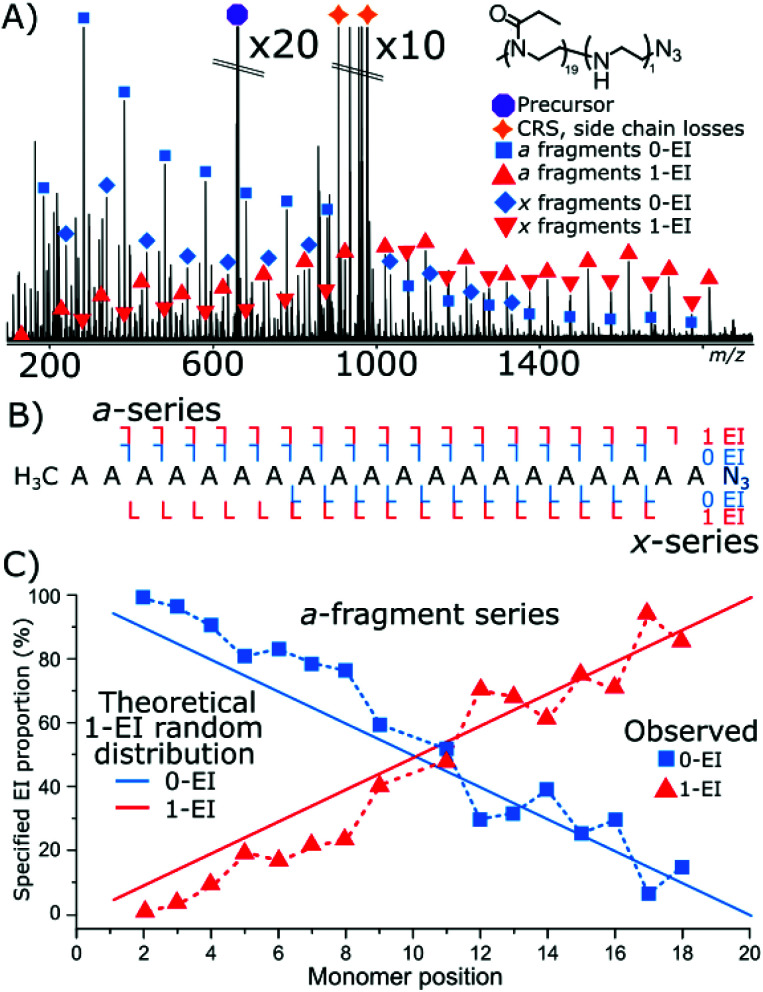
(A) ECD fragmentation of an azide-terminated 20 monomer copolymer species containing 1 EI unit. Main fragment series are highlighted. (B) Fragment coverage diagram (C) plot of EI content on the *a*-series as a function of fragment intensity. And theoretical plot of fragment intensity between the two EI values for a randomly distributed hydrolysis event in the polymer.

Cleavage coverage of 95% was achieved with ECD fragmentation of P(EtOx_19_-*co*-EI_1_)-N_3_ (Fig. 4) showing that the *a*-series consisted of the methyl terminus and two fragment series a 0-EI and 1-EI containing series. The *x*-series contained the N_3_ terminus and 0-EI units and a 1-EI unit series.

**Fig. 5:**
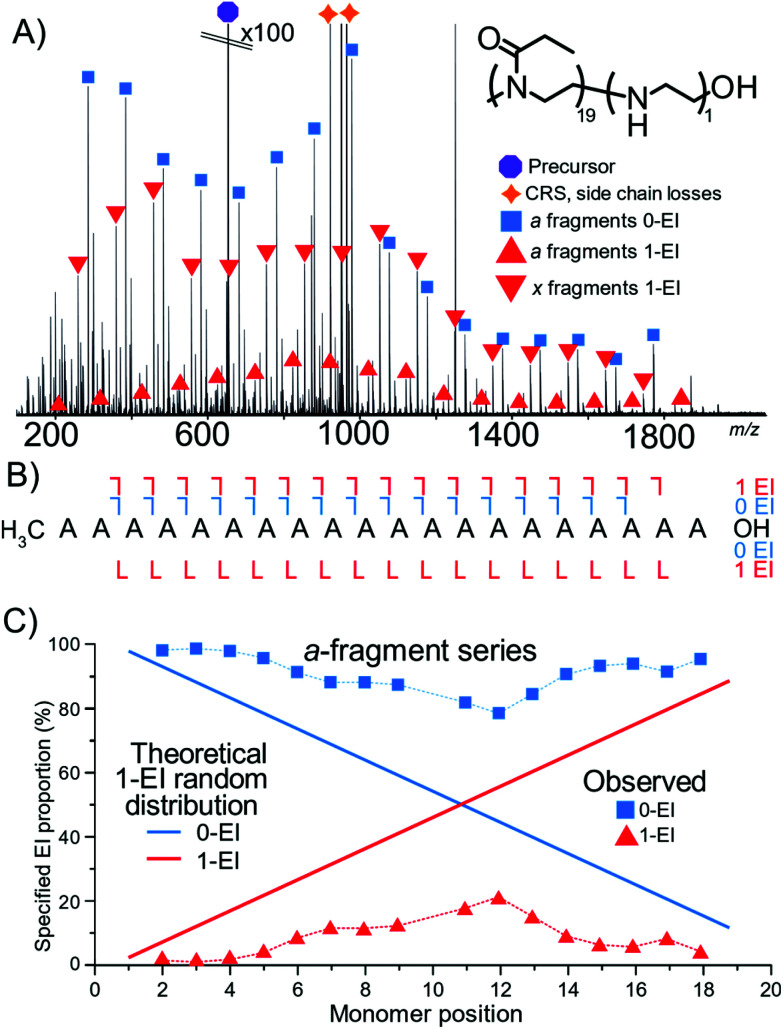
(A) ECD fragmentation of P(EtOx_19_-*co*-EI_1_)-OH (B) fragment coverage diagram, no ω OH terminated 0-EI species are observed. (C) Distribution of the a-series, noting the large deviation from the theoretical plot in Fig. 3D evidence that the hydrolysis is no longer a random process.

Fig. 4C plots the *a*-series fragments, the linearity corresponds to random hydrolysis. The statistical measurements are referenced to the α/methyl terminus the likelihood of observing an EI unit increased linearly towards the N_3_ terminus. Total coverage in the *a* series for 0-EI units was 90% from *a*_2_ (2-POx, *m*/*z* 187.14410) to *a*_18_ (18-Ox, *m*/*z* 1772.24155). The 1-EI series extended from *a*_2_ (1-EtOx1-EI, *m*/*z* 131.11793) to *a*_19_ (18-EtOx1-EI, *m*/*z* 1815.28096) yielding 95% cleavage coverage and 100% sequence coverage. The linear increase in hydrolysis content across the chain is evidence that the hydrolysis process is random with respect to initial monomer position. Changing the ω terminus from an azide (N_3_) to a hydroxyl (OH) functionalisation produced very different behaviour during hydrolysis. Quadrupole isolation of a singly hydrolysed copolymer P(EtOx_19_-*co*-EI_1_)-OH showed no *x*-fragments containing 0-EI units. Only *x*-fragments containing 1-EI unit were observed. Conversely, the *a*-fragment series showed primarily species containing 1-EI unit. The lack of 0-EI oligomer units generated from the OH terminus suggests that hydrolysis occurs in the monomer directly next to the OH terminus at a much higher rate than all other sites (Fig. 5).

The OH terminus increased the level of detected hydrolysis events occurring at the terminal EtOx unit. The same amount of 1-EI containing *a*-series fragments shows that the formation of hydrolysis at any other position in the polymer chain is much less favoured compared to the terminal monomer. The deviation of Fig. 5C compared to Fig. 4D is evidence that the –OH terminus strongly directs the initial hydrolysis event.

**Fig. 6:**
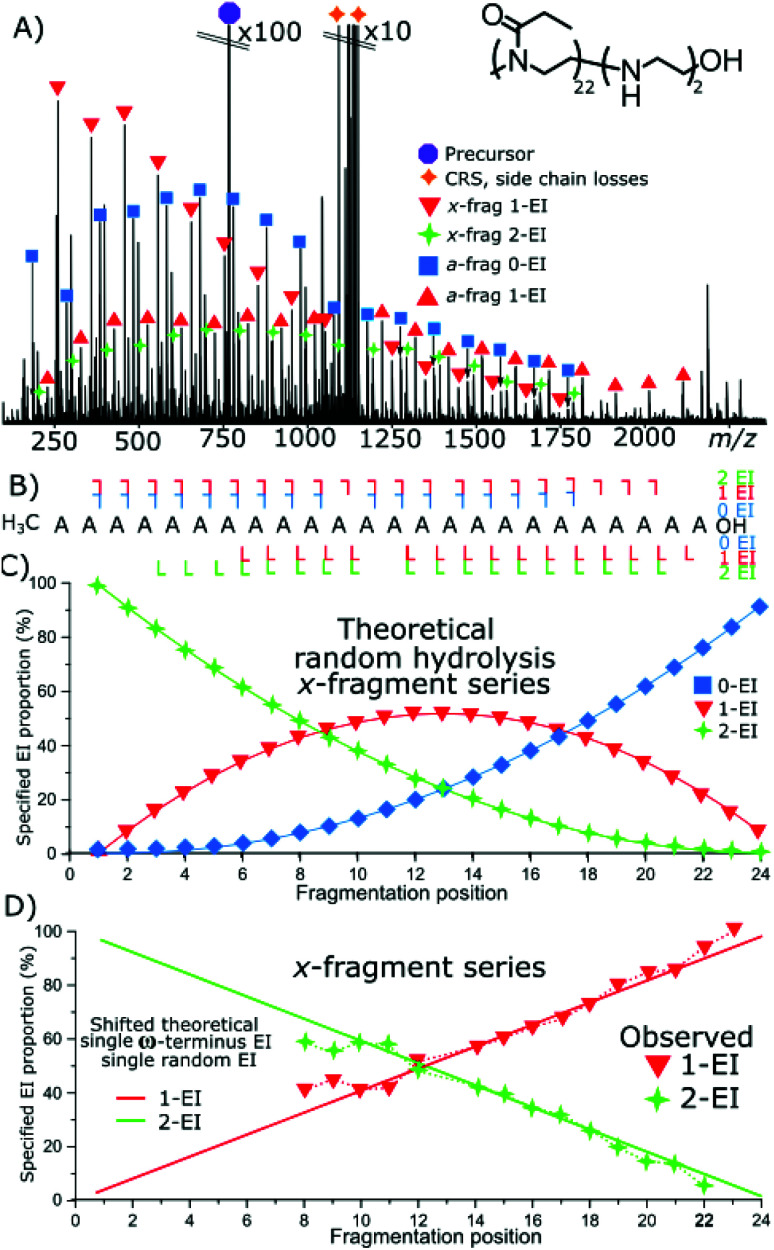
(A) ECD fragmentation of OH-terminated P(EtOx_22_-*co*-EI_2_)-OH species (B) fragment coverage, no OH terminated 0-EI species and 100% sequence coverage of *a*- and *x*-series are observed. (C) Theoretical plot of the *x*-fragment series for random 2 hydrolysis events across the polymer, (D) observed distribution of the EI species of the *x*-fragment series with. “Shifted” theoretical series assuming a hydrolysis event occurring at the ω-terminus and a random hydrolysis process thereafter has a much greater agreement with the observed distribution.

The fragmentation of a doubly modified species, hydroxyl terminated P(EtOx_22_-*co*-EI_2_)-OH copolymer, Fig. 6A, detected hydrolysis events with similar properties to the previous examples. The *x*-series fragmentation consists of 1-EI and 2-EI containing fragments, again, there was no observation of 0-EI containing fragment species. The *a*-series fragments are observed with the expected shift from 0-EI to 1-EI fragments as the fragments increase in size. A small amount of 2-EI *a*-series is observed at very low intensity across the mass spectrum.

Fig. 5C is a theoretical random hydrolysis plot of the expected EI distribution of two EI units across a 24-mer polymer. Notably, the measured result, Fig. 6D, is not close to matching the theoretical distribution. However, by “shifting” the distribution by 1-EI unit, placed on the *x*-terminus, ω/OH, the distribution matches a single EI unit randomly distributed across the polymer species, matching that of the azide terminated species above, Fig. 3, with the presence of 1-EI unit on the OH terminus. Indicating a set modification site at the terminus and a random hydrolysis process thereafter.

Interestingly, with two EI units present the distribution of the other EI unit is unaffected by the consistent terminal hydrolysis event. The result here gives two potential chemical outcomes: the presence of a hydrolysis event does not favour a second hydrolysis event (lack of neighbouring group effect), if this was the case there would be an increased gradient of the loss of 1-EI species and the gain of 2-EI species closer to the terminus. The second is that the hydrolysis event occurring at the terminus engages in a continued interaction which does not affect the hydrolysis rate of neighbouring EtOx units. Neigbouring group effects causing the grouping of hydrolysis events together don't seem to happen within this polymer length.

**Fig. 7:**
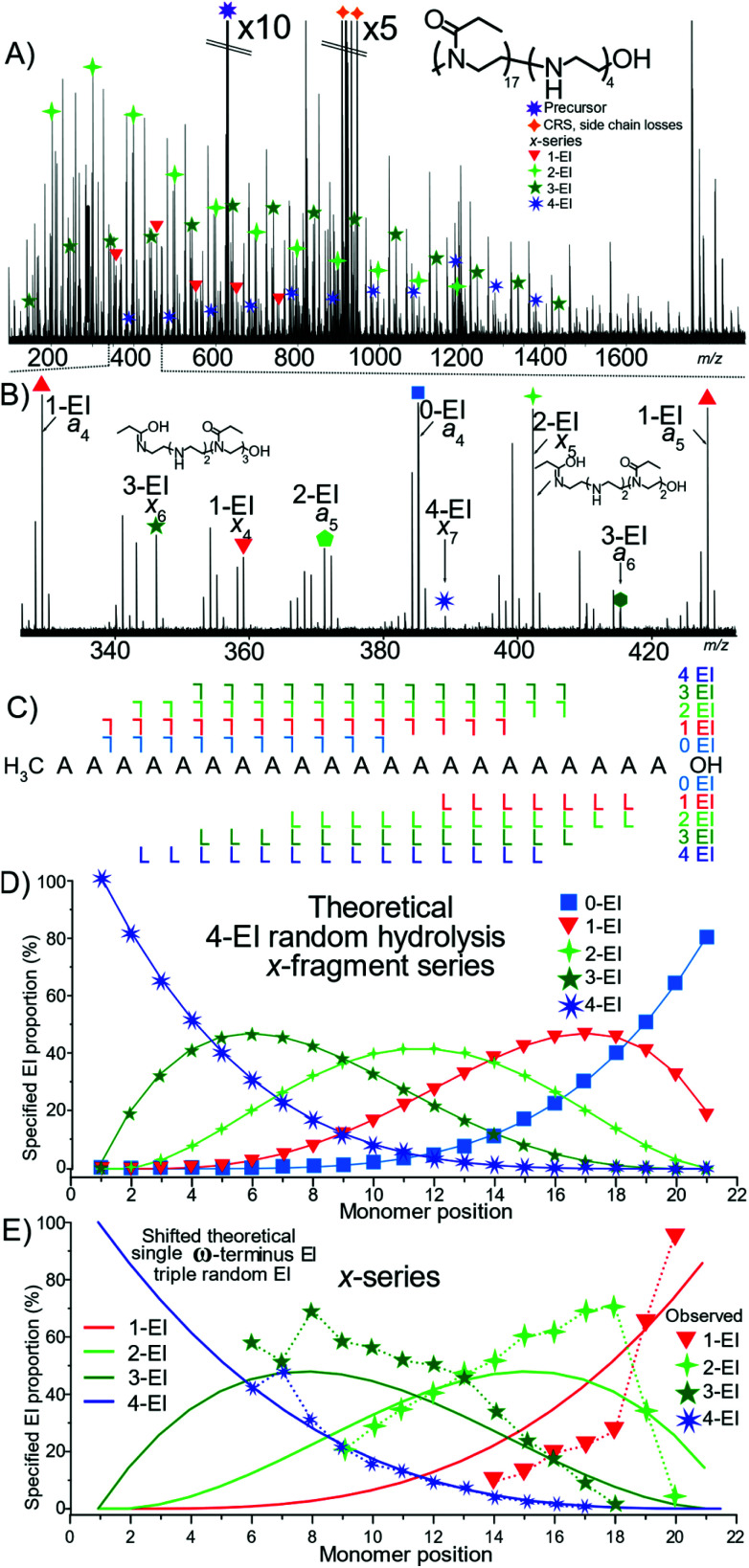
(A) ECD fragmentation of P(EtOx_17_-*co*-EI_4_)-OH only the *x*-series has been annotated here for clarity (B) zoom of region with *a-* and *x-*series fragments annotated (C) Framgnetation diagram with no OH terminated 0-EI species observed. (D) Theoretical *x*-series plot based on randomised hydrolysis process. (E) Fragment proportion of fragment hydrolysis distribution and “shifted” theoretical series assuming a hydrolysis event has occurred at the ω-terminus and a random process thereafter has a much greater agreement with the observed distribution.

Analysis of a tetra-modified species P(EtOx_17_-*co*-EI_4_)-OH polymer by ECD MS/MS produced extensive structural information, Fig. 7A. The different levels of EI *a* and *x* containing fragments, and corresponding internal fragments increase the complexity significantly requiring high resolving power and mass accuracy. Fig. 7C presents the fragmentation map of the hydrolysed polymer. The presence of many 4-EI oligomers in the *x*-series and no detected 0-EI oligomers indicated that hydrolysis occurred preferentially at the OH terminus.

Fig. 7D is the theoretical plot of 4 hydrolysed monomers within a 21-monomer polymer below it, Fig. 7E, the measured data has numerous fragment types but does not closely align with the theoretical plot. The data closely aligns with that of a 4 hydrolysed species with a single hydrolysis event at the terminus, Fig. 7F. Comparing Fig. 7E and F shows the presence of single hydrolysis event at the terminus, but then very little significant change of the randomness after the initial hydrolysis event. Again, the lack of neighboring group effect is displayed here.

The results presented in Fig. 4–6 allow some conclusions to be made about how the hydrolysis of the polyoxazoline occurs:

The ω-terminated OH causes a terminus directing hydrolysis effect: the data presented in Fig. 4 shows that the hydrolysis events were evenly spread over the polyoxazoline chain with an N3 terminating group. Changing the N3 group for a OH terminating group, Fig. 5, causes most of the hydrolysis to occur on the polyoxazoline unit adjacent to the OH terminus, removing the random hydrolysis effect. The presence of the constant hydrolysis next to the OH terminus suggests an activations effect, potentially through hydrogen bonding, of the polyoxazoline monomer adjacent to the terminus.

A significant neighbouring group effect is not shown after a terminus hydrolysis event: The presence of a significant neighbouring group effect, one in which the hydrolysis of a polyoxazoline unit increases the favourability of adjacent hydrolysis events, would be observed if hydrolysis is directed in one place causes further hydrolysis at the adjacent polyoxazoline groups. A neighbouring group effect would be observed in the proportion plots as being an area of much higher gradient around a directed hydrolysis event. Fig. 5 shows that the even with a hydrolysis directed to one terminus of the molecule the second hydrolysis event did not occur in a biased fashion, either at the neighbouring polyoxazoline unit to the initial hydrolysis event, nor any other position. The second hydrolysis event is randomly distributed through the polyoxazoline chain suggesting that, in this case, a neighbouring group effect did not occur.

## Conclusion

In conclusion, we have shown that mass spectrometry offers unique data for the analysis of partially modified co-polymer species, and illustrated this approach to provide an in-depth characterization of poly[(oxazoline)-*co*-(ethylenimine)], a novel gene delivery vector. Relative monomer ratios across polymer species may be measured by MS giving a unique advantage over NMR. The presence of an OH terminus group was found to produce a hydrolysed monomer directly adjacent to the terminus. Further hydrolysis after the terminal hydrolysis was randomly distributed showing no neighbouring group effect, and that hydrolysis remains stochastic. While this data shows random polymerisation over these chain lengths, and cannot be explicitly extended to longer chain lengths, it is reasonable to expect that this stochastic effect is independent of chain length, until more evidence becomes available. The knowledge of how hydrolysed (charged) groups are distributed within the P(EtOx-*co*-EI) vector is key to assess and understand their gene delivery properties and toxicity. Furthermore, beyond these specific materials, the information and methods presented in this study can be used for any number of end groups, and polymer structures to test whether polymer modification is random, synergistic, or at discrete favoured positions, and may be further extended to any biological or synthetic molecule that undergoes a random modification. The ability to ascertain synthetic properties of polymer modifications shown here, is of great use to the highly controlled needs of synthetic medicinal chemists.

## Author contributions

T. M. and T. F. conceived and planned experiments, T. M. B. M. and C. W. performed, developed, and designed analytical and statistical methods. T. F. synthesised all polymer species. M. B. contributed instrumentation support, and verification of analytical methods. A. B. S. P. and P. O'C. proposed and supervised the project. All authors were involved in manuscript editing, authoring, and checking the final manuscript.

## Conflicts of interest

There are no conflict of interests to declare.

## Supplementary Material

PY-013-D2PY00437B-s001
