# Supplementary material for: Stochasticity of poly(2-oxazoline) oligomer hydrolysis determined by tandem mass spectrometry
Source: Polym Chem. 2022 Jun 2;13(28):4162–9. doi: 10.1039/d2py00437b (PMC9294869; doi:10.1039/d2py00437b)
Supplement: PY-013-D2PY00437B-s001 [file PY-013-D2PY00437B-s001.pdf]

Electronic Supplementary Material (ESI) for Polymer Chemistry.
This journal is © The Royal Society of Chemistry 2022

SUPPORTING INFORMATION

to

Stochasticity of poly(2-oxazoline) oligomer hydrolysis determined by tandem mass spectrometry

by

Tomos E. Morgan[1], Thomas Floyd[1], Bryan Marzullo[1], Christopher A. Wootton[1], , Mark P. Barrow[1], Anthony W. T. Bristow[2], Sebastien Perrier[1], Peter B. O'Connor[1]*

[1]Department of Chemistry, University of Warwick, Coventry, Midlands, CV4 7AL, UK.

[2]Chemical Development, Pharmaceutical Technology & Development, Operations, AstraZeneca, Macclesfield, UK.

*Corresponding authors: Peter O'Connor p.oconnor@warwick.ac.uk

Contents

Section S1: Mass spectrometry conditions 2

Section S2: Additional analysis notes 2

Section S2: Theoretical plotting of random distributions 3

Section S3: Synthesis of Poly(oxazoline) and species 3

Section S4: hydrolysis of poly(2-oxazoline) 6

Table S 1: Pre-determined heating time in microwave reactor and corresponding hydrolysis level of a poly(2-oxazoline) 6

Section S5: Characterisation of poly(2-oxazoline) 6

Section S6: Characterisation of P(Ox-*co*-EI) species 8

Figure S 1: nESI Mass spectrum of POx before hydrolysis 12

Figure S 1: nESI Mass spectrum of POx before hydrolysis 12

Table S 2: MS assignment of the POx 13

Table S 3: MS assignment of P($Ox_{75\%}$-*co*-$EI_{25\%}$)-OH Figure 2A in main text 15

Table S 4: MS assignment of P($Ox_{75\%}$-*co*-$EI_{25\%}$)-N_3 Figure 2C in main text 18

Table S 5: ECD assignment of P(Ox_{19}-*co*-EI_1)-N_3 Figure 3A in main text 22

Figure S 2: *x*-series fragmentation diagram P(Ox_{19}-*co*-EI_1)-N_3 reverse of Figure 3C in text 24

Table S 7: ECD fragmentation assignment of a p(22Pox-*r*-2EI)OH Figure 5 in main text 28

Table S 8: ECD assignment of P(Ox_{17}-*co*-EI_4)-OH Figure 5A 30

Section S7: Generation of combinations Python 3 34

Section S1: Mass spectrometry conditions

The hydrolysed sample was dissolved into purified water obtained from a Direct-Q3 Ultrapure Water System (Millipore, Lutterworth, United Kingdom) at 20 µM and acidified for analysis via addition of 0.5% formic acid (v/v) (Sigma-Aldrich, Dorset, United Kingdom). All experiments were performed on a 12 T solariX Fourier transform ion cyclotron resonance mass spectrometer (Bruker Daltonik, GmbH, Bremen, Germany) using a nano-electrospray (nESI) ion source in positive-ion mode. The ECD was carried out with the use of an indirectly heated hollow cathode with a current set at 1.5 A, with a pulse length of 0.2 s and bias 1.2 V. All data were recorded using 4 mega-word (2^{22}, 22 bit) transients (1.6777 s) achieving approximately 500,000 resolving power at *m/z* 400 for the intact mass spectrometry with a mass cut off at *m/z* 147 and 400,000 resolving power at *m/z* 400 for the tandem mass spectrometry with a low mass cut off at *m/z* 100. All mass spectra were internally calibrated by the intact polymer peaks across the polymer distribution, or by internal calibration of fragment peaks in ECD spectra (peaks used for calibration are marked). The peaks used for internal calibration were crosschecked using both the *a* and *x* fragment series. The Bruker SNAP algorithm was used for peak picking with the polyoxazoline monomer used as the repeat unit (C_5H_9NO). The Bruker SNAP algorithm matches a calculated isotope distribution adjusted to a repeat unit with increasing mass. [1]

Section S2: Additional analysis notes

Figure 3: The proportions represented in Figure 3 are the fragment intensities comparing the peak area of the 0-EI containing fragment and the 1-EI containing fragment at each monomer position. By calculating the total peak area of fragments at each monomer position the relative proportions of differing EI amounts can be compared to one another generating a plot, Figure 3C. The theoretical plot, Figure 3D, assumes completely random hydrolysis, calculated using the same method discussed in the experimental but with a single hydrolysis event randomly distributed across a 20-monomer species. Deviation from the theoretical plots indicates deviation from completely random hydrolysis events during the synthetic process.

Figure 3C shows the analysis comparing the area of each modified/unmodified peak pair. The total areas of the a_n peaks in both the 0-EI and 1-EI series were summed at the ratio between the two compared. The results, presented in Figure 3, closely align with the theoretical plot, Figure 3D. Showing the presence of the 1-EI group trending upwards linearly across the length of the polymer chain.

Figure 6: ***x*-series fragments:** The *x*-series fragments consisted of 1-EI to 4-EI containing oligomer fragments. The 1-EI species were present from x_2 to x_8 (*m/z* 161.12847, 0.05 ppm; *m/z* 854.60736, -0.03 ppm respectively). 2-EI species were observed from x_2 (*m/z* 105.10229, 0.48 ppm) to x_{13} (*m/z* 1194.8543, -0.41 ppm). 3-EI oligomers were present from x_4 to x_{18} (*m/z* 148.14447, 0.21 ppm; *m/z* 1535.10141, -0.50 ppm respectively), and the maximum hydrolysis level 4-EI oligomers were observed from x_5 (*m/z* 191.18667, 0.16 ppm) to x_{16} (*m/z* 1380.00773, 0.1 ppm).

***a*-series fragments:** Conversely the *a*-series fragments consisted of a 0-EI containing fragment series from a_2 to a_{11}, (*m/z* 187.14410, -0.08 ppm; *m/z* 1177.82781, 0.02 ppm respectively). The 1-EI containing oligomers were present from x_2 (*m/z* 131.11792, 0.23 ppm) to x_{16} (*m/z* 1518.07434, -0.88 ppm). The 2-EI species from a_3 to a_{18} (*m/z* 174.16009, -0.2 ppm; *m/z* 1660.18170, -2.7 ppm). The final hydrolysis level observed was 3-EI containing species consisting of a_5 (*m/z* 316.27076, 0.18 ppm) to a_{17} (*m/z* 1604.15787, -1.37 ppm).

The 0-EI *a*-series was observed from a_2 to a_{19} (*m/z* 187.14410, 0.03 ppm and *m/z* 1871.30715, -0.1 ppm respectively), 1-EI containing species were present from a_3 to a_{22} (*m/z* 230.18630, -0.02 ppm to *m/z* 2112.49051, 2.0 ppm). The *x* series has a 1-EI containing series from x_2 to x_{18} (*m/z* 161.12848, 0.16

ppm, *m/z* 1746.22161, -0.84 ppm. The presence of 2-EI containing fragments were present from x_3 to x_{17} (*m/z* 204.17065, -0.02 ppm and *m/z* 1591.1284, 0.06 ppm).

Section S2: Theoretical plotting of random distributions

The use of tandem mass spectrometry to localize non-specific modification positions graphically has been effectively carried out using DNA,[2] we extend this by predicting and then fitting to, random distributions. The fragmentation data was compared to the statistically distributed fragmentation patterns. The statistically distributed hydrolysis maps were calculated by combination of PEI units within a polymer chain using a modified Heap's algorithm.[3] The total number of arrangements was calculated and the fragment intensities were calculated by code included in the SI. Figure 1 shows a theoretical model of 2 EI units evenly distributed across five monomer units using the Heap's algorithm and how, at different fragmentation points, the total proportion of each species will vary. Put simply:

Random hydrolysis events (H) will evenly distribute across all possible combinations. All possible combinations will be statistically represented during the analysis.

At monomer position **1** measuring back to the α (left) methyl terminus 40% of fragments have one hydrolysis event (H) as only one monomer unit is present; a doubly hydrolysed species can't be present. The remaining 60% of fragments possible have not undergone a hydrolysis event. One hydrolysis event (H) represents the presence of and EI species. Depending on whether the fragment contains 0, 1, or 2 hydrolysis events (H) dictate whether that fragment is a 0-EI, 1-EI, or 2-EI containing species respectively.

Moving to monomer position **2** 60% of measured fragment oligomers contain one hydrolysis event (H). 30% of fragments contain no hydrolysis events and 10% of fragments contained 2 hydrolysis events.

Fragmentation at each monomer and the resulting oligomer unit can be analyzed in the same way and the proportions compared.

If the practical data shows similar binomial distribution to the theoretical plot then they hydrolysis is random, if there is a large shift in the distribution then it is not random.

Practically, the peak areas at each monomer position are compared. For example, the 0-EI a_3, 1-EI a_3, and 2-EI a_3 fragment peak areas are compared to one another. The peak area is calculated within the DataAnalysis program and the same peak picking is used for all assignments. As the measurement is relative to other peaks in a given summed spectrum, deviations in signal to noise from spectrum to spectrum do not influence the techniques use, and fragments are similar enough in abundance and resolved well enough that S/N variation has little effect on individual monomer positions.

Section S3: Synthesis of Poly(oxazoline) and species

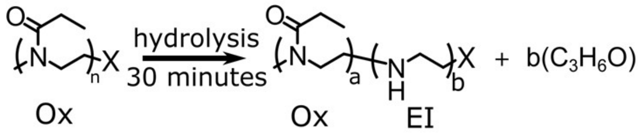

Scheme S1: Overview of synthesis of P(Ox-*co*-EI)-OH, through hydrolysis of POx.

Materials

2-ethyl-2-oxazoline (EtOx, > 99.9%, Sigma-Aldrich) was distilled over barium oxide prior to use. methyl *p*-toluenesulfonate (MeTos, 98 %, Alfa Aesar) was distilled prior to use. acetonitrile extra dry (99.9%, Acros Organics), tetramethylammonium hydroxide solution (25 wt % in methanol, Acros Organics) and hydrochloric acid (HCl, 37 %, Fisher Scientific) were used as received.

Instrumentation

Size Exclusion Chromatography

P(EtOx)-OH was measured on an Agilent Infinity II MDS instrument equipped with differential refractive index (DRI), viscometry (VS), dual angle light scatter (LS) and multiple wavelength UV detectors. The system was equipped with 2 x PLgel Mixed C columns (300 x 7.5 mm) and a PLgel 5 µm guard column. The eluent is $CHCl_3$ with 2 % TEA (triethylamine). Samples were run at 1 ml min^{-1} at 30 °C. Poly(methyl methacrylate), and polystyrene standards (Agilent EasyVials) were used for calibration. Ethanol was added as a flow rate marker.

P(EtOx)-N_3 was measured on an An Agilent Infinity II MDS instrument equipped with differential refractive index (DRI), viscometry (VS), dual angle light scatter (LS) and multiple wavelength UV detectors was used for SEC analysis. The system was fitted with 2 x PLgel Mixed D columns (300 x 7.5 mm) and a PLgel 5 µm guard column. The eluent used was DMF with 5 mmol NH_4BH_4 additive. Samples were run at 1 ml min^{-1} at 50 °C. Poly(methyl methacrylate) standards (Agilent EasyVials) were used for calibration between 955,500 – 550 g mol^{-1}.

Analyte samples were filtered through a GVHP membrane with 0.22 µm pore size before injection. Respectively, experimental molar mass ($M_{n,\ SEC}$) and dispersity ($Đ$) values of synthesized polymers were determined by conventional calibration using Agilent GPC/SEC software.

Nuclear Magnetic Resonance

Proton nuclear magnetic resonance spectra (^1H NMR) were recorded on a Bruker Advance 300 spectrometer (300 MHz), with chemical shift values (δ) reported in ppm, and the residual proton signal of the solvent used as internal standard. ^1H NMR of P(EtOx) homopolymers was measured in $CDCl_3$. ^1H NMR of P(EtOx-*co*-EI) copolymers was measured in CD_3OD

Synthesis

Synthesis of ω-hydroxyl-poly(2-ethyl-2-oxazoline) (pEtOx-OH)

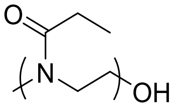

Under a stream of N_2, EtOx (3.965 g, 4.00 x 10^{-2} mol), MeTos (0.372 g, 2 x 10^{-3} mol) and Acetonitrile (5.66 mL) were transferred to a Schlenk flask that had been placed in a 150 °C oven overnight. The Schlenk flask was sealed and placed into an oil bath, preheated to 80 °C. After 2 hr, the Schlenk flask was opened under a stream of N_2 and tetramethylammonium Hydroxide solution (1.094 g, 3 x 10^{-3} mol) was added to terminate the polymerisation. The reaction mixture was left overnight to ensure complete termination before removal of volatiles by rotary evaporation. The isolated polymer was dissolved in chloroform and washed with saturated sodium carbonate (x 3) and brine (x 3). The polymer was precipitated in diethyl ether (x 3) and dried under vacuum to yield a white solid. ^{1}H NMR (400 MHz, $CDCl_3$, 298 K) δ (ppm): 3.8 – 3.1 (m, 80 H, backbone), 3.1 – 2.9 (m, 3 H, methyl (α-end group)), 2.5 – 2.1 (m, 40 H, CH_2 side chain) 1.2 – 0.9 (m, 60 H, CH_3 side chain). SEC ($CHCl_3$ + 2 % TEA): M_w = 2,600 g mol^{-1}, $Đ$ = 1.16.

Synthesis of ω-azido-poly(2-ethyl-2-oxazoline) (pEtOx-N_3)

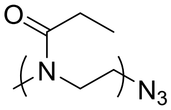

Under a stream of N_2, EtOx (3.965 g, 4.00 x 10^{-2} mol), MeTos (0.372 g, 2 x 10^{-3} mol) and acetonitrile (5.66 mL) were transferred to a Schlenk flask that had been placed in a 150 °C oven overnight. The Schlenk flask was sealed and placed into an oil bath, preheated to 80 °C. After 2 hr, the Schlenk flask was opened under a stream of N_2 and sodium azide (0.600 g, 9.23 x 10^{-3} mol) was added to terminate the polymerisation. The reaction mixture was left overnight to ensure complete termination before removal of volatiles by rotary evaporation. The isolated polymer was dissolved in chloroform and washed with saturated sodium carbonate (x 3) and brine (x 3). The polymer was precipitated in diethyl ether (x 3) and dried under vacuum to yield a white solid. ^{1}H NMR (400 MHz, $CDCl_3$, 298 K) δ (ppm): 3.8 – 3.1 (m, 80 H, backbone), 3.1 – 2.9 (m, 3 H, methyl (α-end group)), 2.5 – 2.1 (m, 40 H, CH_2 side chain) 1.2 – 0.9 (m, 60 H, CH_3 side chain). SEC (DMF + 5 mmol NH_4BH_4): M_w = 4,000 g mol^{-1}, $Đ$ = 1.08.

Section S4: hydrolysis of poly(2-oxazoline)

Hydrolysis of poly(2-ethyl-2-oxazoline)

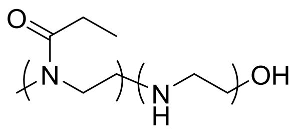

pEtOx (0.08 g, [Amide] = 0.48 M) was dissolved in deionised water (1.54 mL) and transferred to a Biotage microwave reactor vial (0.5 – 2 mL) along with HCl (0.14 mL, [HCl] = 1.0 M). The vial was sealed and placed into a Biotage Initiator+ Eight microwave reactor and heated to 120 °C for a pre-determined time (see **Table S 1**). Once complete, NaOH solution (0.555 mL, [NaOH] = 4.0 M) was added to neutralise the solution, followed by dialysis against deionised water (500 – 1000 Da MWCO). The polymer was then isolated by freeze drying. ^{1}H NMR (400 MHz, MeOD, 298 K) δ (ppm): 3.8 – 3.4 (m, oxazoline backbone), 3.15 – 3.05 (m, methyl (α-end group)), 2.9 – 2.7 (m, ethylenimine backbone) 2.6 – 2.3 (m, CH_2 side chain) 1.2 – 1.0 (m, CH_3 side chain).

The degree of hydrolysis determined by ^{1}H-NMR was calculated using the integration values (I) and **Equation S1**

$$Hydrolysis\ (\%) = \frac{I\ [pEI\ Backbone]}{I\ [pEI\ Backbone] + I\ [pEtOx\ Backbone]}\ x\ 100$$

Equation S1: Calculation of total hydrolysis as a % of pEI content.

Table S 1: Pre-determined heating time in microwave reactor and corresponding hydrolysis level of a poly(2-oxazoline)

**Polymer**	**Time (min)**	**Hydrolysis (%)**
$P(EtOx)_{20}$-OH	5	10.0
$P(EtOx)_{20}$-OH	10	10.0
$P(EtOx)_{20}$-OH	20	18.0
$P(EtOx)_{20}$-OH	30	25.0
$P(EtOx)_{20}$-N_3	30	20.0

Section S5: Characterisation of poly(2-oxazoline)

Characterisation

Nuclear Magnetic Resonance (NMR)

P(EtOx)$_{20}$-OH

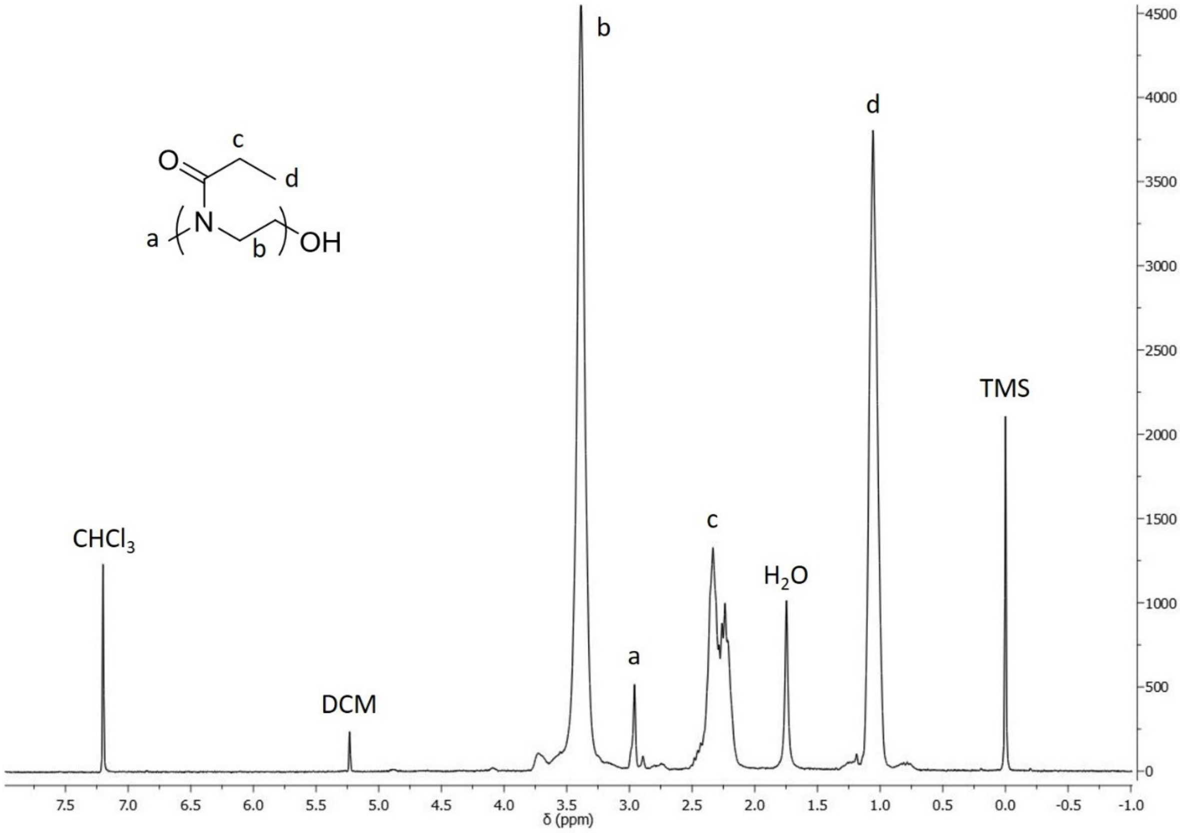

Size Exclusion Chromatography

P(EtOx)$_{20}$-OH

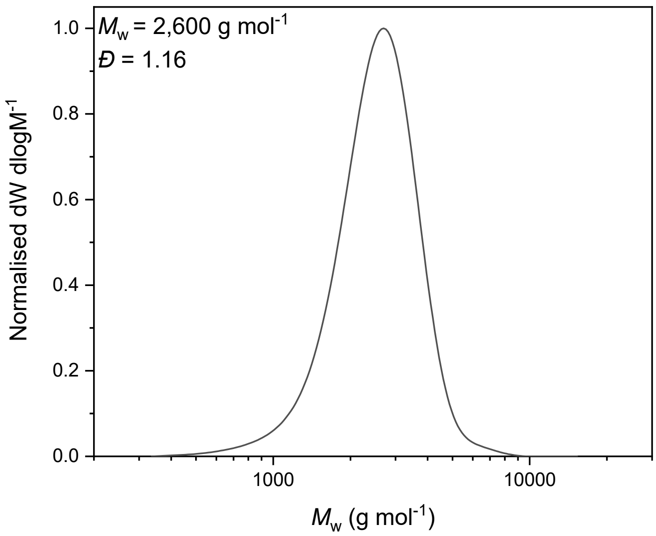

P(EtOx)$_{20}$-N$_3$

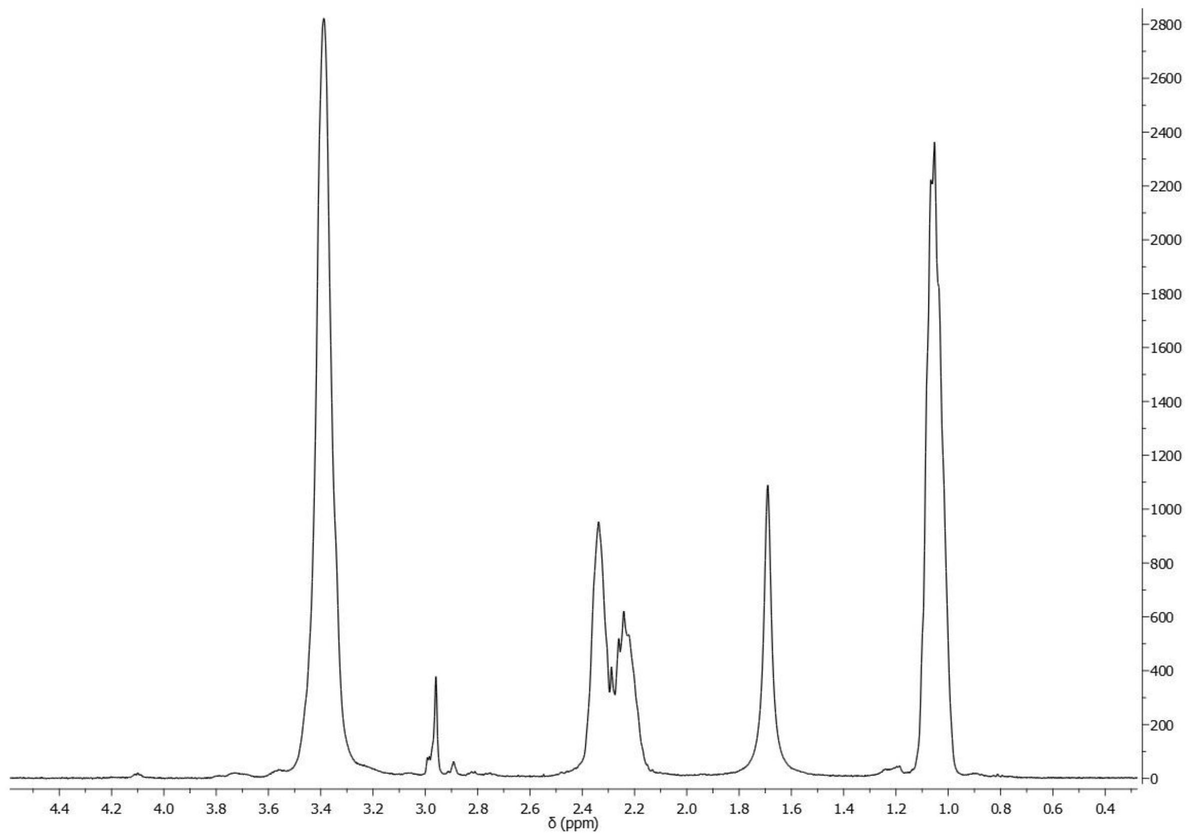

P(EtOx)$_{20}$-N$_3$

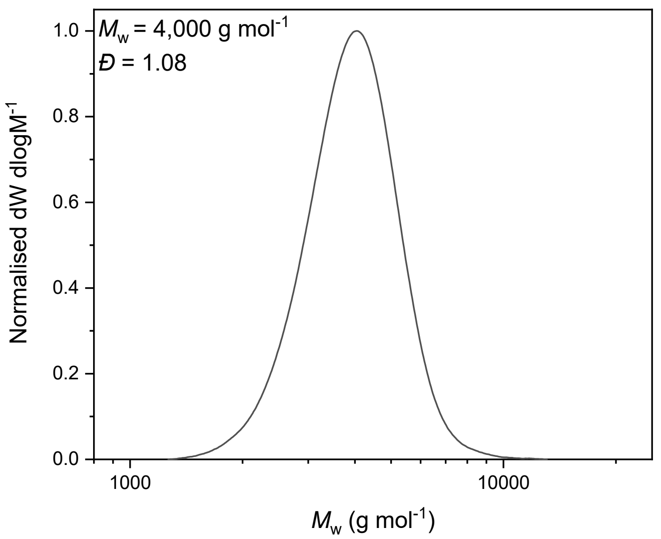

Section S6: Characterisation of P(Ox-*co*-EI) species

Nuclear Magnetic Resonance (NMR)

P(Ox$_{0.9}$-*co*-EI$_{0.1}$)-OH

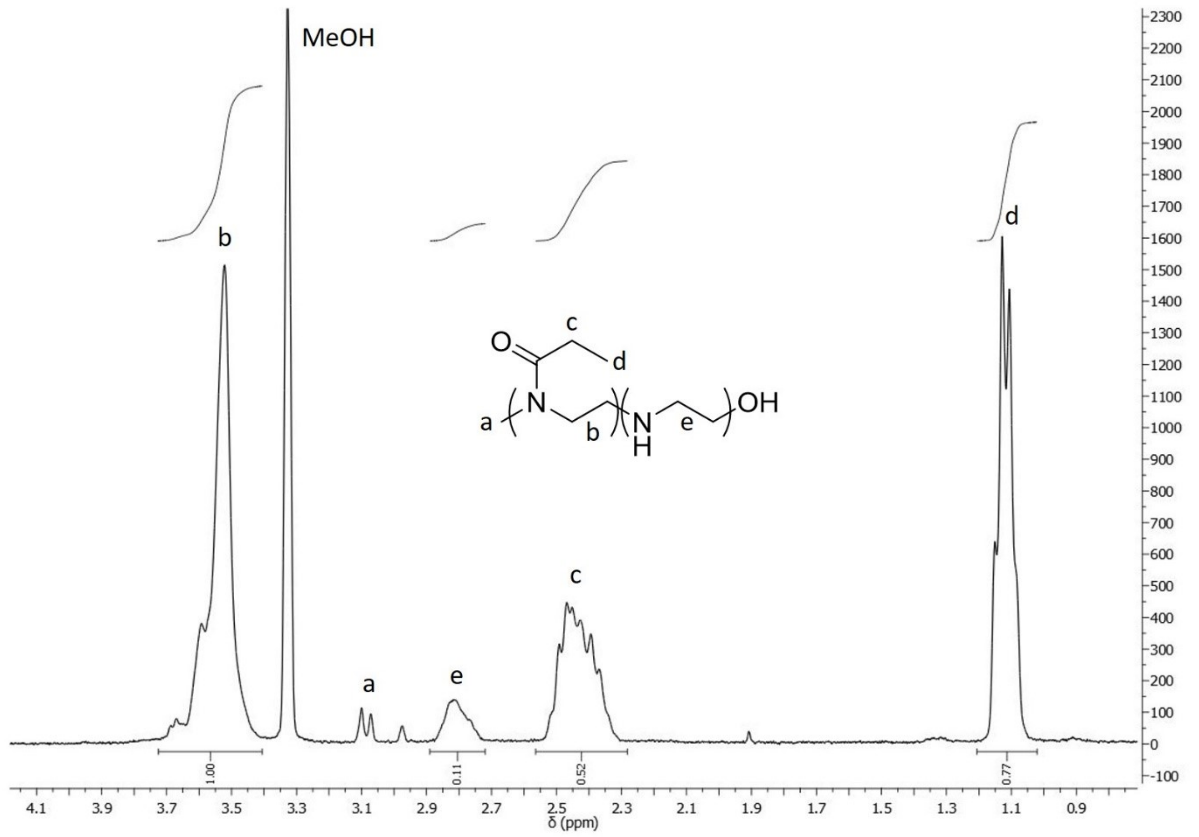

P(Ox$_{0.9}$-*co*-EI$_{0.1}$)-OH

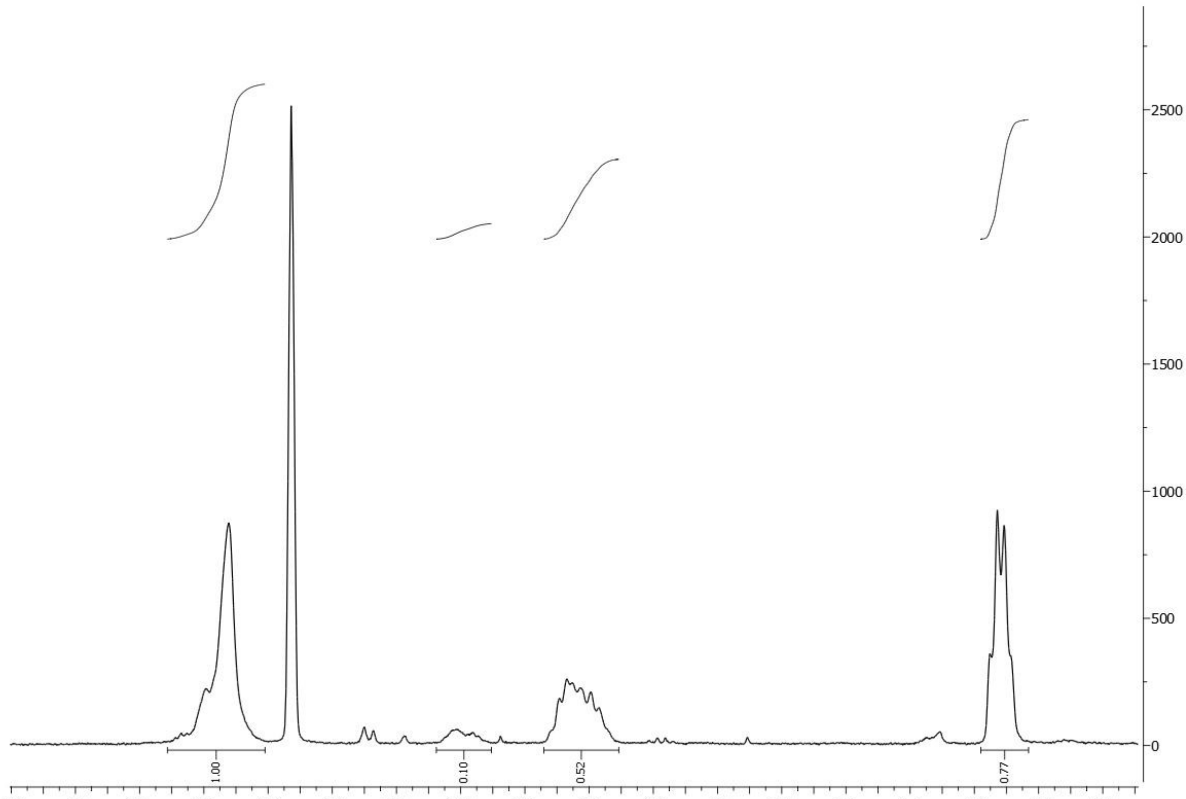

P(Ox$_{0.82}$-*co*-EI$_{0.18}$)-OH

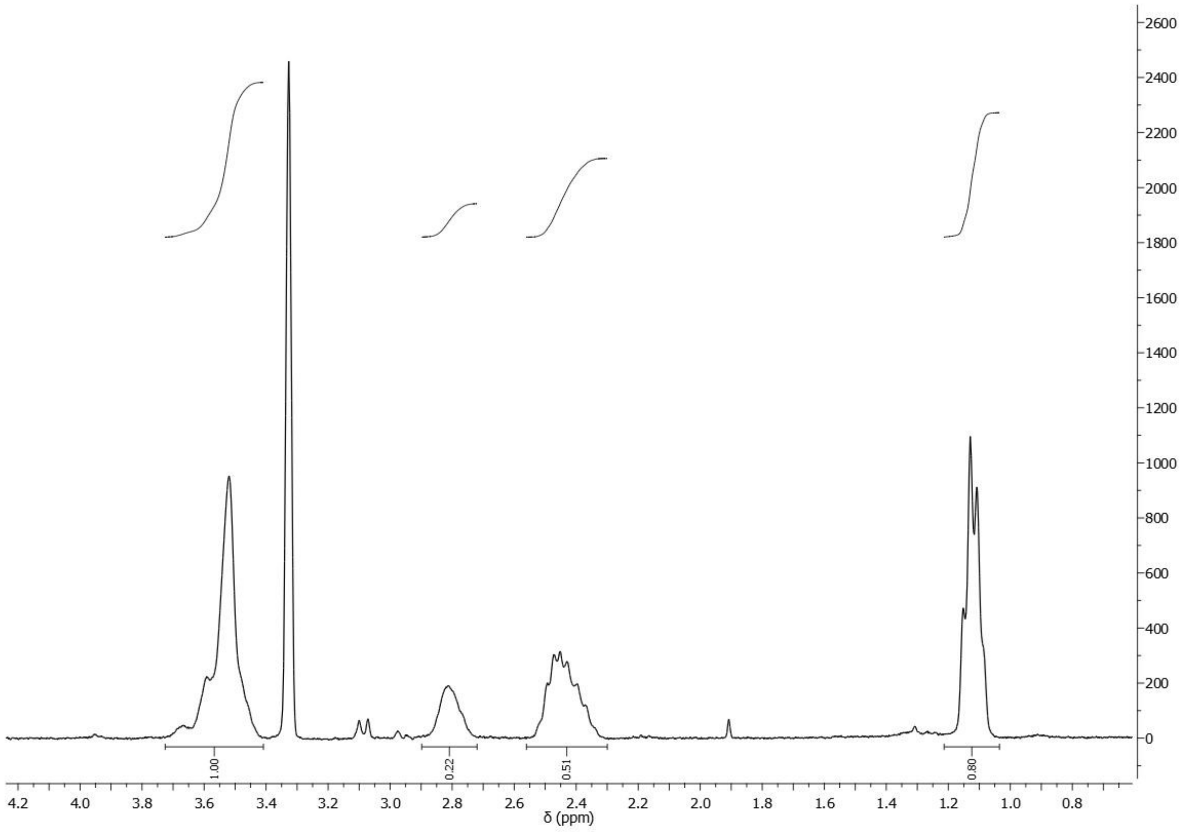

P(Ox$_{0.75}$-*co*-EI$_{0.25}$)-OH

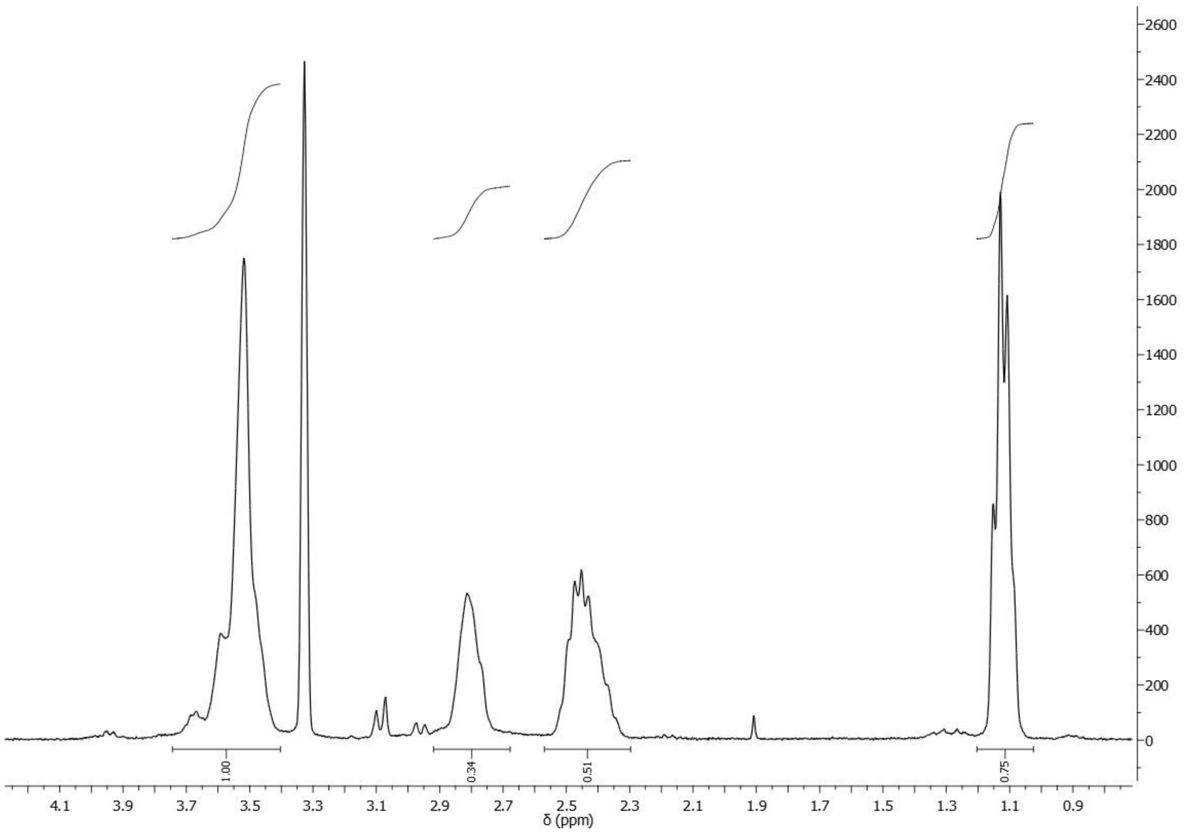

P(Ox$_{0.80}$-*co*-EI$_{0.20}$)-N$_3$

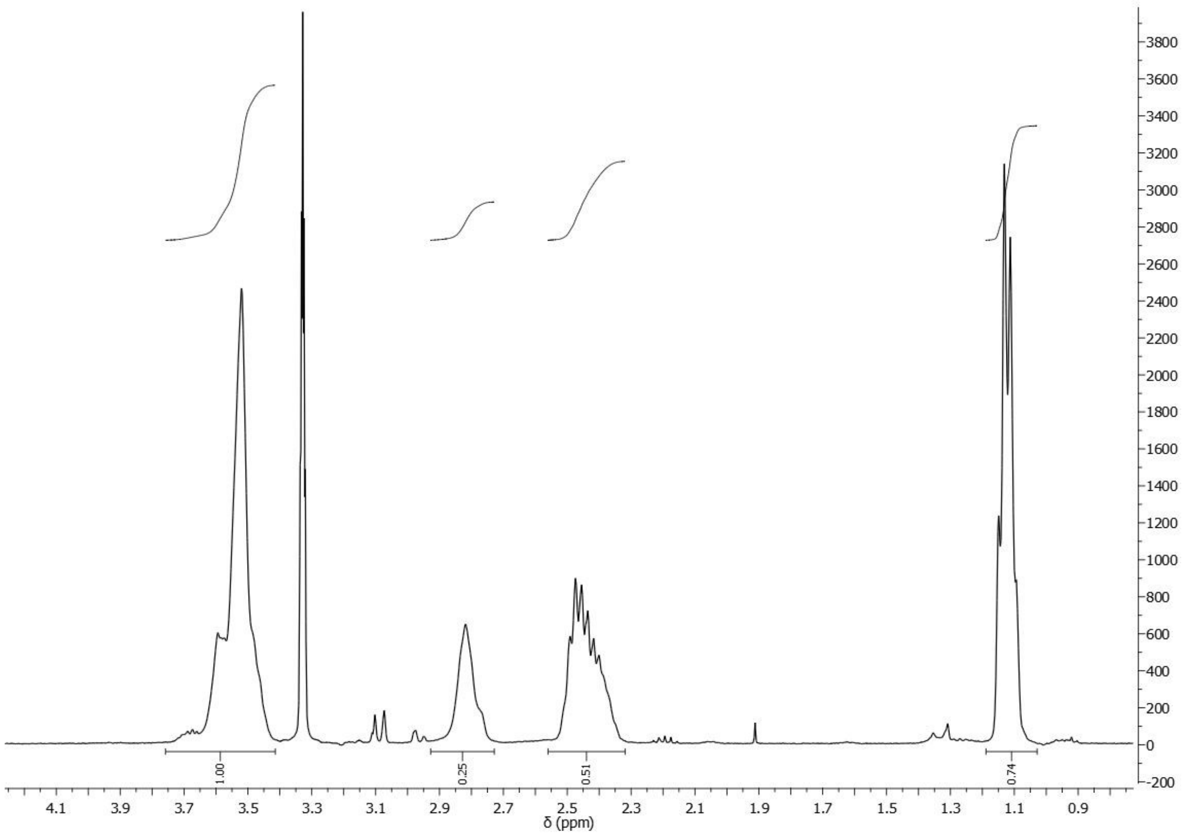

1.00
0.25
0.51
0.74
δ (ppm)

Figure S 1: nESI Mass spectrum of POx before hydrolysis

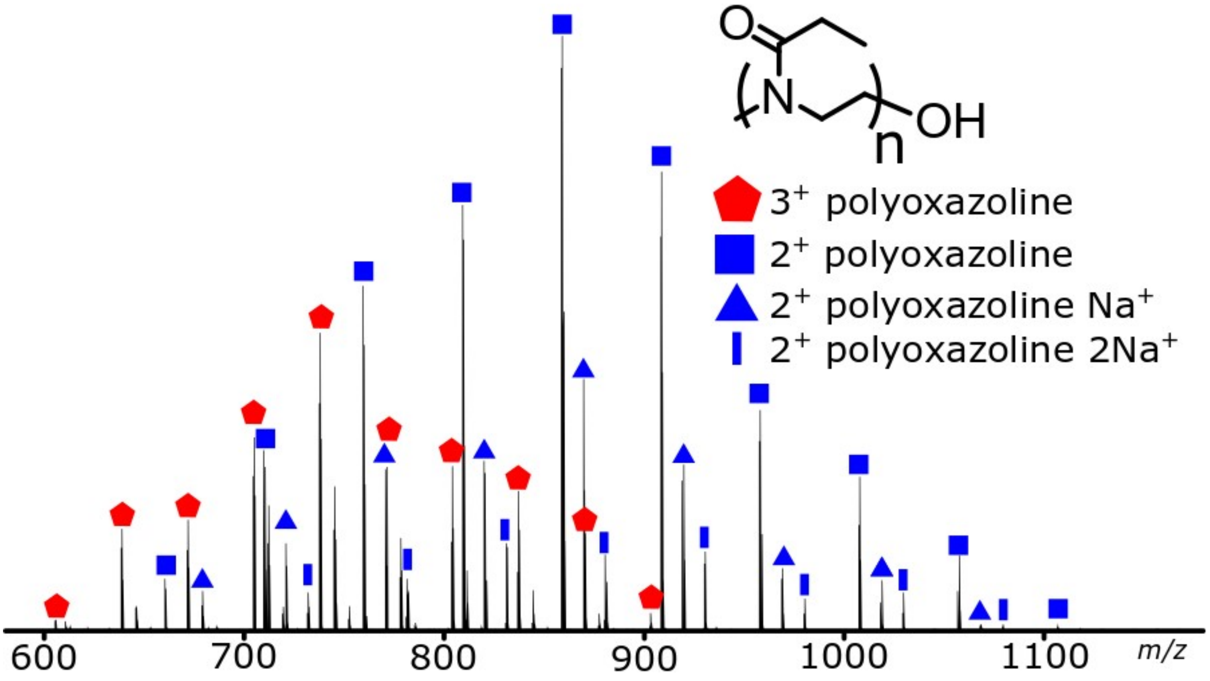

Figure S 2: nESI Mass spectrum of POx before hydrolysis

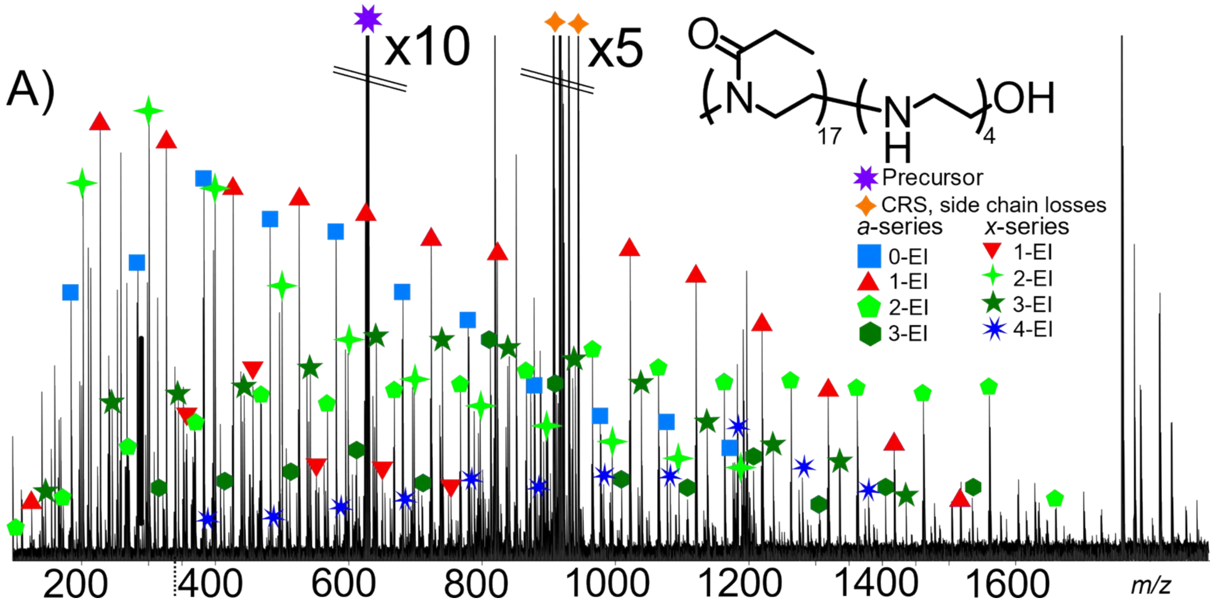

Annotated figure 7 showing both *a* and *x*-series fragments.

Table S 2: MS assignment of the POx

**m/z**	**z**	**Chemical formula assigned**	**Formula**	**error (ppm)**
561.8967	2	$C_{56}H_{103}N_{11}O_{12}H+_{2}$	p(EtOx$_{11}$)	0.00
611.4313	2	$C_{61}H_{112}N_{12}O_{13}H+_{2}$	p(EtOx$_{12}$)	0.64
660.9655	2	$C_{66}H_{121}N_{13}O_{14}H+_{2}$	p(EtOx$_{13}$)	0.66
710.4993	2	$C_{71}H_{130}N_{14}O_{15}H+_{2}$	p(EtOx$_{14}$)	0.03
760.0335	2	$C_{76}H_{139}N_{15}O_{16}H+_{2}$	p(EtOx$_{15}$)	-0.01
809.5669	2	$C_{81}H_{148}N_{16}O_{17}H+_{2}$	p(EtOx$_{16}$)	-0.93
859.1011	2	$C_{86}H_{157}N_{17}O_{18}H+_{2}$	p(EtOx$_{17}$)	-0.95
908.6361	2	$C_{91}H_{166}N_{18}O_{19}H+_{2}$	p(EtOx$_{18}$)	-0.04
958.1704	2	$C_{96}H_{175}N_{19}O_{20}H+_{2}$	p(EtOx$_{19}$)	0.12
1007.707	2	$C_{101}H_{184}N_{20}O_{21}H+_{2}$	p(EtOx$_{20}$)	2.12
1057.241	2	$C_{106}H_{193}N_{21}O_{22}H+_{2}$	p(EtOx$_{21}$)	2.53
1106.773	2	$C_{111}H_{202}N_{22}O_{23}H+_{2}$	p(EtOx$_{22}$)	0.02
1156.306	2	$C_{116}H_{211}N_{23}O_{24}H+_{2}$	p(EtOx$_{23}$)	-1.21
1205.84	2	$C_{121}H_{220}N_{24}O_{25}H+_{2}$	p(EtOx$_{24}$)	-1.00
613.4209	3	$C_{91}H_{166}N_{18}O_{19}H+_{2}Na+_{1}$	p(EtOx$_{18}$)	0.40
646.4439	3	$C_{96}H_{175}N_{19}O_{20}H+_{2}Na+_{1}$	p(EtOx$_{19}$)	0.69
679.4662	3	$C_{101}H_{184}N_{20}O_{21}H+_{2}Na+_{1}$	p(EtOx$_{20}$)	-0.05
712.4886	3	$C_{106}H_{193}N_{21}O_{22}H+_{2}Na+_{1}$	p(EtOx$_{21}$)	-0.72
745.5118	3	$C_{111}H_{202}N_{22}O_{23}H+_{2}Na+_{1}$	p(EtOx$_{22}$)	-0.15
778.5342	3	$C_{116}H_{211}N_{23}O_{24}H+_{2}Na+_{1}$	p(EtOx$_{23}$)	-0.61
811.5566	3	$C_{121}H_{220}N_{24}O_{25}H+_{2}Na+_{1}$	p(EtOx$_{24}$)	-1.15
844.5815	3	$C_{126}H_{229}N_{25}O_{26}H+_{2}Na+_{1}$	p(EtOx$_{25}$)	1.39
877.6049	3	$C_{131}H_{238}N_{26}O_{27}H+_{2}Na+_{1}$	p(EtOx$_{26}$)	2.02
633.4124	2	$C_{61}H_{112}N_{12}O_{13}Na+_{2}$	p(EtOx$_{12}$)	-1.47
682.946	2	$C_{66}H_{121}N_{13}O_{14}Na+_{2}$	p(EtOx$_{13}$)	-2.31
732.4809	2	$C_{71}H_{130}N_{14}O_{15}Na+_{2}$	p(EtOx$_{14}$)	-1.21
782.0153	2	$C_{76}H_{139}N_{15}O_{16}Na+_{2}$	p(EtOx$_{15}$)	-0.82
831.5489	2	$C_{81}H_{148}N_{16}O_{17}Na+_{2}$	p(EtOx$_{16}$)	-1.60
881.0853	2	$C_{86}H_{157}N_{17}O_{18}Na+_{2}$	p(EtOx$_{17}$)	1.07
930.6174	2	$C_{91}H_{166}N_{18}O_{19}Na+_{2}$	p(EtOx$_{18}$)	-1.28
980.1495	2	$C_{96}H_{175}N_{19}O_{20}Na+_{2}$	p(EtOx$_{19}$)	-3.43
1029.685	2	$C_{101}H_{184}N_{20}O_{21}Na+_{2}$	p(EtOx$_{20}$)	-1.93
1079.219	2	$C_{106}H_{193}N_{21}O_{22}Na+_{2}$	p(EtOx$_{21}$)	-1.74
1128.754	2	$C_{111}H_{202}N_{22}O_{23}Na+_{2}$	p(EtOx$_{22}$)	-1.20
1178.29	2	$C_{116}H_{211}N_{23}O_{24}Na+_{2}$	p(EtOx$_{23}$)	0.20
1227.825	2	$C_{121}H_{220}N_{24}O_{25}Na+_{2}$	p(EtOx$_{24}$)	0.71
919.6286	2	$C_{91}H_{166}N_{18}O_{19}H+_{1}Na+_{1}$	p(EtOx$_{18}$)	1.35
969.1641	2	$C_{96}H_{175}N_{19}O_{20}H+_{1}Na+_{1}$	p(EtOx$_{19}$)	2.59
1018.699	2	$C_{101}H_{184}N_{20}O_{21}H+_{1}Na+_{1}$	p(EtOx$_{20}$)	3.51
1117.767	2	$C_{111}H_{202}N_{22}O_{23}H+_{1}Na+_{1}$	p(EtOx$_{21}$)	2.26
620.7471	3	$C_{91}H_{166}N_{18}O_{19}H+_{1}Na+_{2}$	p(EtOx$_{22}$)	-1.76
653.7703	3	$C_{96}H_{175}N_{19}O_{20}H+_{1}Na+_{2}$	p(EtOx$_{23}$)	-1.02
686.7932	3	$C_{101}H_{184}N_{20}O_{21}H+_{1}Na+_{2}$	p(EtOx$_{24}$)	-0.84
719.8162	3	$C_{106}H_{193}N_{21}O_{22}H+_{1}Na+_{2}$	p(EtOx$_{25}$)	-0.53

752.8389	3	$C_{111}H_{202}N_{22}O_{23}H+_{1}Na+_{2}$	p(EtOx$_{26}$)	-0.61
785.8606	3	$C_{116}H_{211}N_{23}O_{24}H+_{1}Na+_{2}$	p(EtOx$_{27}$)	-1.99
818.8833	3	$C_{121}H_{220}N_{24}O_{25}H+_{1}Na+_{2}$	p(EtOx$_{28}$)	-2.09
851.9055	3	$C_{126}H_{229}N_{25}O_{26}H+_{1}Na+_{2}$	p(EtOx$_{29}$)	-2.74
884.9284	3	$C_{131}H_{238}N_{26}O_{27}H+_{1}Na+_{2}$	p(EtOx$_{30}$)	-2.54
		Average Error (ppm)		1.23
		Standard deviation (ppm)		1.31

Table S 3: MS assignment of P(Ox$_{75\%}$-*co*-EI$_{25\%}$)-OH Figure 2A in main text

m/z	charge	Pox	PEI	chemical formula	error	area
531.39202	3	14	4	$C_{79}H_{150}N_{18}O_{15}H^+_3$	0.89	32556
564.4145	3	15	4	$C_{84}H_{159}N_{19}O_{16}H^+_3$	0.27	252229
597.43731	3	16	4	$C_{89}H_{168}N_{20}O_{17}H^+_3$	0.26	744042
630.46014	3	17	4	$C_{94}H_{177}N_{21}O_{18}H^+_3$	0.29	1611874
663.4829	3	18	4	$C_{99}H_{186}N_{22}O_{19}H^+_3$	0.21	1172455
696.50622	3	19	4	$C_{104}H_{195}N_{23}O_{20}H^+_3$	0.94	1038064
729.52893	3	20	4	$C_{109}H_{204}N_{24}O_{21}H^+_3$	0.76	777592
762.55132	3	21	4	$C_{114}H_{213}N_{25}O_{22}H^+_3$	0.19	163225
795.574	3	22	4	$C_{119}H_{222}N_{26}O_{23}H^+_3$	0.02	40505
591.43319	3	15	4	$C_{89}H_{166}N_{20}O_{16}H^+_3$	-0.75	23945
624.45609	3	16	4	$C_{94}H_{175}N_{21}O_{17}H^+_3$	-0.56	95570
657.47839	3	17	4	$C_{99}H_{184}N_{22}O_{18}H^+_3$	-1.30	134700
690.50133	3	18	4	$C_{104}H_{193}N_{23}O_{19}H^+_3$	-1.04	67374
669.50093	2	11	5	$C_{66}H_{128}N_{16}O_{12}H^+_2$	-1.83	10744
719.03834	2	12	5	$C_{71}H_{137}N_{17}O_{13}H^+_2$	2.75	14354
768.56906	2	13	5	$C_{76}H_{146}N_{18}O_{14}H^+_2$	-1.97	39222
818.10297	2	14	5	$C_{81}H_{155}N_{19}O_{15}H^+_2$	-2.21	29967
867.64426	2	15	5	$C_{86}H_{164}N_{20}O_{16}H^+_2$	6.08	40470
917.17618	2	16	5	$C_{91}H_{173}N_{21}O_{17}H^+_2$	3.26	36302
545.73892	3	14	5	$C_{81}H_{155}N_{19}O_{15}H^+_3$	-0.05	103531
578.76179	3	15	5	$C_{86}H_{164}N_{20}O_{16}H^+_3$	0.07	524084
611.78461	3	16	5	$C_{91}H_{173}N_{21}O_{17}H^+_3$	0.09	927185
644.80738	3	17	5	$C_{96}H_{182}N_{22}O_{18}H^+_3$	0.03	1366747
677.8301	3	18	5	$C_{101}H_{191}N_{23}O_{19}H^+_3$	-0.09	655737
710.85242	3	19	5	$C_{106}H_{200}N_{24}O_{20}H^+_3$	-0.77	765933
743.87662	3	20	5	$C_{111}H_{209}N_{25}O_{21}H^+_3$	1.14	87662
704.0068	2	9	2	$C_{70}H_{131}N_{15}O_{14}H^+_2$	-0.67	481861
753.54251	2	10	2	$C_{75}H_{140}N_{16}O_{15}H^+_2$	1.37	733586
803.07649	2	11	2	$C_{80}H_{149}N_{17}O_{16}H^+_2$	1.00	860961
852.60957	2	12	2	$C_{85}H_{158}N_{18}O_{17}H^+_2$	-0.38	594528
902.14495	2	13	2	$C_{90}H_{167}N_{19}O_{18}H^+_2$	0.94	199016
951.67931	2	14	2	$C_{95}H_{176}N_{20}O_{19}H^+_2$	1.05	45720
675.99343	2	12	3	$C_{67}H_{127}N_{15}O_{13}H^+_2$	-1.09	133985
725.52877	2	13	3	$C_{72}H_{136}N_{16}O_{14}H^+_2$	0.55	870220
775.0626	2	14	3	$C_{77}H_{145}N_{17}O_{15}H^+_2$	0.03	774867
824.5986	2	15	3	$C_{82}H_{154}N_{18}O_{16}H^+_2$	2.20	262327
874.13361	2	16	3	$C_{87}H_{163}N_{19}O_{17}H^+_2$	2.99	359251
923.66472	2	17	3	$C_{92}H_{172}N_{20}O_{18}H^+_2$	-0.52	66105
550.06689	3	16	3	$C_{82}H_{154}N_{18}O_{16}H^+_3$	-0.11	20980
616.11272	3	17	3	$C_{92}H_{172}N_{20}O_{18}H^+_3$	0.26	471137
649.13559	3	18	3	$C_{97}H_{181}N_{21}O_{19}H^+_3$	0.35	741345
682.15857	3	19	3	$C_{102}H_{190}N_{22}O_{20}H^+_3$	0.59	389137
715.18145	3	20	3	$C_{107}H_{199}N_{23}O_{21}H^+_3$	0.67	558522
601.45281	3	14	7	$C_{90}H_{172}N_{22}O_{15}H^+_3$	-0.36	31366
634.47553	3	15	7	$C_{95}H_{181}N_{23}O_{16}H^+_3$	-0.48	96855

667.49811	3	16	7	$C_{100}H_{190}N_{24}O_{17}H^{+}_{3}$	-0.79	20275
700.52024	3	17	7	$C_{105}H_{199}N_{25}O_{18}H^{+}_{3}$	-1.71	31739
733.54346	3	18	7	$C_{110}H_{208}N_{26}O_{19}H^{+}_{3}$	-1.07	17758
697.51418	2	13	4	$C_{69}H_{132}N_{16}O_{13}H^{+}_{2}$	-1.56	41579
796.5814	2	14	4	$C_{79}H_{150}N_{18}O_{15}H^{+}_{2}$	-2.86	58447
846.1203	2	15	4	$C_{84}H_{159}N_{19}O_{16}H^{+}_{2}$	2.85	80179
895.65248	2	16	4	$C_{89}H_{168}N_{20}O_{17}H^{+}_{2}$	0.43	77660
945.18603	2	17	4	$C_{94}H_{177}N_{21}O_{18}H^{+}_{2}$	-0.29	54768
560.08635	3	14	6	$C_{83}H_{160}N_{20}O_{15}H^{+}_{3}$	0.01	107125
593.10908	3	15	6	$C_{88}H_{169}N_{21}O_{16}H^{+}_{3}$	-0.12	371318
626.13174	3	16	6	$C_{93}H_{178}N_{22}O_{17}H^{+}_{3}$	-0.34	738401
659.15459	3	17	6	$C_{98}H_{187}N_{23}O_{18}H^{+}_{3}$	-0.26	412014
692.17698	3	18	6	$C_{103}H_{196}N_{24}O_{19}H^{+}_{3}$	-0.84	173682
758.22199	3	19	6	$C_{113}H_{214}N_{26}O_{21}H^{+}_{3}$	-1.56	39683
574.43385	3	14	7	$C_{85}H_{165}N_{21}O_{15}H^{+}_{3}$	0.18	81219
607.4566	3	15	7	$C_{90}H_{174}N_{22}O_{16}H^{+}_{3}$	0.08	118453
640.47921	3	16	7	$C_{95}H_{183}N_{23}O_{17}H^{+}_{3}$	-0.22	239348
673.50183	3	17	7	$C_{100}H_{192}N_{24}O_{18}H^{+}_{3}$	-0.49	33577
706.52647	3	18	7	$C_{105}H_{201}N_{25}O_{19}H^{+}_{3}$	2.13	29888
739.54723	3	19	7	$C_{110}H_{210}N_{26}O_{20}H^{+}_{3}$	-0.73	8516
519.38538	4	18	6	$C_{103}H_{196}N_{24}O_{19}H^{+}_{4}$	0.75	21388
544.15215	4	19	6	$C_{108}H_{205}N_{25}O_{20}H^{+}_{4}$	0.10	110539
568.91923	4	20	6	$C_{113}H_{214}N_{26}O_{21}H^{+}_{4}$	0.06	149344
593.68627	4	21	6	$C_{118}H_{223}N_{27}O_{22}H^{+}_{4}$	-0.05	111628
618.45373	4	22	6	$C_{123}H_{232}N_{28}O_{23}H^{+}_{4}$	0.52	57899
643.21939	4	23	6	$C_{128}H_{241}N_{29}O_{24}H^{+}_{4}$	-1.74	34332
587.10544	3	14	6	$C_{88}H_{167}N_{21}O_{15}H^{+}_{3}$	-0.32	20425
620.12845	3	15	6	$C_{93}H_{176}N_{22}O_{16}H^{+}_{3}$	0.03	136030
653.15086	3	16	6	$C_{98}H_{185}N_{23}O_{17}H^{+}_{3}$	-0.58	164385
686.17328	3	17	6	$C_{103}H_{194}N_{24}O_{18}H^{+}_{3}$	-1.11	59136
719.19522	3	18	6	$C_{108}H_{203}N_{25}O_{19}H^{+}_{3}$	-2.26	27738
861.14651	2	14	7	$C_{85}H_{165}N_{21}O_{15}H^{+}_{2}$	-0.54	19639
621.80311	3	15	8	$C_{92}H_{179}N_{23}O_{16}H^{+}_{3}$	-1.35	26412
533.39189	4	19	5	$C_{106}H_{200}N_{24}O_{20}H^{+}_{4}$	0.65	63464
558.15881	4	20	5	$C_{111}H_{209}N_{25}O_{21}H^{+}_{4}$	0.29	88228
607.69245	4	22	5	$C_{121}H_{227}N_{27}O_{23}H^{+}_{4}$	-0.67	57746
632.45936	4	23	5	$C_{126}H_{236}N_{28}O_{24}H^{+}_{4}$	-0.95	80369
572.75773	3	14	5	$C_{86}H_{162}N_{20}O_{15}H^{+}_{3}$	-0.87	29696
605.78086	3	15	5	$C_{91}H_{171}N_{21}O_{16}H^{+}_{3}$	-0.28	96267
638.80398	3	16	5	$C_{96}H_{180}N_{22}O_{17}H^{+}_{3}$	0.22	412849
671.82665	3	17	5	$C_{101}H_{189}N_{23}O_{18}H^{+}_{3}$	0.01	112816
704.84953	3	18	5	$C_{106}H_{198}N_{24}O_{19}H^{+}_{3}$	0.12	145144
737.87214	3	19	5	$C_{111}H_{207}N_{25}O_{20}H^{+}_{3}$	-0.15	55128
770.89441	3	20	5	$C_{116}H_{216}N_{26}O_{21}H^{+}_{3}$	-0.84	24756
505.37859	4	17	7	$C_{100}H_{192}N_{24}O_{18}H^{+}_{4}$	0.30	8835
530.14579	4	18	7	$C_{105}H_{201}N_{25}O_{19}H^{+}_{4}$	0.47	67299
554.91268	4	19	7	$C_{110}H_{210}N_{26}O_{20}H^{+}_{4}$	0.06	66539

579.67986	4	20	7	$C_{115}H_{219}N_{27}O_{21}H^{+}_{4}$	0.19	101139
604.44696	4	21	7	$C_{120}H_{228}N_{28}O_{22}H^{+}_{4}$	0.18	39485
516.13936	4	17	8	$C_{102}H_{197}N_{25}O_{18}H^{+}_{4}$	0.72	7406
540.90622	4	18	8	$C_{107}H_{206}N_{26}O_{19}H^{+}_{4}$	0.24	43703
565.67288	4	19	8	$C_{112}H_{215}N_{27}O_{20}H^{+}_{4}$	-0.56	36012
590.44029	4	20	8	$C_{117}H_{224}N_{28}O_{21}H^{+}_{4}$	-0.01	28392
				Average (ppm)	0.01	
				Standard deviation (ppm)	1.23	
				Sum weighted EI (ppm)	99113345	
				Sum weighted Ox (ppm)	384740786	
				Ratio	25.76%	

Table S 4: MS assignment of P(Ox$_{75\%}$-*co*-EI$_{25\%}$)-N$_3$ Figure 2C in main text

m/z	charge	Pox	PEI	chemical formula	error	area	OH/N$_3$
562.72908	3	15	1	$C_{83}H_{152}N_{20}O_{16}H^+_3$	-2.43	15989	N_3
595.75285	3	16	1	$C_{88}H_{161}N_{21}O_{17}H^+_3$	-0.68	140335	N_3
628.77448	3	17	1	$C_{93}H_{170}N_{22}O_{18}H^+_3$	-2.51	122452	N_3
661.79912	3	18	1	$C_{98}H_{179}N_{23}O_{19}H^+_3$	0.39	97772	N_3
727.84634	3	20	1	$C_{108}H_{197}N_{25}O_{21}H^+_3$	2.57	12386	N_3
478.0082	3	12	2	$C_{70}H_{130}N_{18}O_{13}H^+_3$	-2.58	2001	N_3
511.03185	3	13	2	$C_{75}H_{139}N_{19}O_{14}H^+_3$	-0.76	42133	N_3
544.05481	3	14	2	$C_{80}H_{148}N_{20}O_{15}H^+_3$	-0.43	143444	N_3
577.07793	3	15	2	$C_{85}H_{157}N_{21}O_{16}H^+_3$	0.14	424300	N_3
610.1002	3	16	2	$C_{90}H_{166}N_{22}O_{17}H^+_3$	-0.74	749273	N_3
643.12318	3	17	2	$C_{95}H_{175}N_{23}O_{18}H^+_3$	-0.43	761914	N_3
676.14667	3	18	2	$C_{100}H_{184}N_{24}O_{19}H^+_3$	0.60	665552	N_3
709.16868	3	19	2	$C_{105}H_{193}N_{25}O_{20}H^+_3$	-0.55	469230	N_3
742.19033	3	20	2	$C_{110}H_{202}N_{26}O_{21}H^+_3$	-2.08	187834	N_3
775.21184	3	21	2	$C_{115}H_{211}N_{27}O_{22}H^+_3$	-3.66	10535	N_3
808.23529	3	22	2	$C_{120}H_{220}N_{28}O_{23}H^+_3$	-2.71	8248	N_3
459.33448	3	11	3	$C_{67}H_{126}N_{18}O_{12}H^+_3$	0.98	21219	N_3
492.35682	3	12	3	$C_{72}H_{135}N_{19}O_{13}H^+_3$	-0.03	169770	N_3
525.37949	3	13	3	$C_{77}H_{144}N_{20}O_{14}H^+_3$	-0.28	393136	N_3
591.426	3	15	3	$C_{87}H_{162}N_{22}O_{16}H^+_3$	1.27	273630	N_3
624.4478	3	16	3	$C_{92}H_{171}N_{23}O_{17}H^+_3$	-0.41	994471	N_3
657.47081	3	17	3	$C_{97}H_{180}N_{24}O_{18}H^+_3$	-0.07	1153729	N_3
690.49364	3	18	3	$C_{102}H_{189}N_{25}O_{19}H^+_3$	-0.03	1120444	N_3
723.51648	3	19	3	$C_{107}H_{198}N_{26}O_{20}H^+_3$	0.02	785368	N_3
756.53814	3	20	3	$C_{112}H_{207}N_{27}O_{21}H^+_3$	-1.50	255919	N_3
789.5645	3	21	3	$C_{117}H_{216}N_{28}O_{22}H^+_3$	3.07	45949	N_3
822.58535	3	22	3	$C_{122}H_{225}N_{29}O_{23}H^+_3$	0.57	8628	N_3
440.65862	3	11	4	$C_{64}H_{122}N_{18}O_{11}H^+_3$	-0.01	16252	N_3
473.68101	3	12	4	$C_{69}H_{131}N_{19}O_{12}H^+_3$	-0.89	82051	N_3
506.70414	3	13	4	$C_{74}H_{140}N_{20}O_{13}H^+_3$	-0.19	189057	N_3
539.72699	3	14	4	$C_{79}H_{149}N_{21}O_{14}H^+_3$	-0.09	385973	N_3
572.74948	3	15	4	$C_{84}H_{158}N_{22}O_{15}H^+_3$	-0.64	543764	N_3
605.77273	3	16	4	$C_{89}H_{167}N_{23}O_{16}H^+_3$	0.13	583174	N_3
638.7959	3	17	4	$C_{94}H_{176}N_{24}O_{17}H^+_3$	0.70	679353	N_3
671.81887	3	18	4	$C_{99}H_{185}N_{25}O_{18}H^+_3$	0.91	334933	N_3
704.84216	3	19	4	$C_{104}H_{194}N_{26}O_{19}H^+_3$	1.56	126435	N_3
770.88645	3	21	4	$C_{114}H_{212}N_{28}O_{21}H^+_3$	-0.29	10420	N_3
488.02839	3	11	5	$C_{71}H_{136}N_{20}O_{12}H^+_3$	-0.90	56846	N_3
521.05133	3	12	5	$C_{76}H_{145}N_{21}O_{13}H^+_3$	-0.58	109164	N_3
554.07312	3	13	5	$C_{81}H_{154}N_{22}O_{14}H^+_3$	-2.38	209474	N_3
587.09678	3	14	5	$C_{86}H_{163}N_{23}O_{15}H^+_3$	-0.79	175804	N_3
620.11944	3	15	5	$C_{91}H_{172}N_{24}O_{16}H^+_3$	-0.98	117266	N_3
686.16392	3	16	5	$C_{101}H_{190}N_{26}O_{18}H^+_3$	-2.53	14875	N_3
617.44206	2	10	2	$C_{60}H_{112}N_{16}O_{11}H^+_2$	-0.07	288410	N_3
666.97659	2	11	2	$C_{65}H_{121}N_{17}O_{12}H^+_2$	0.42	428372	N_3

716.51093	2	12	2	$C_{70}H_{130}N_{18}O_{13}H^{+}_{2}$	0.58	548131	N_3
766.04432	2	13	2	$C_{75}H_{139}N_{19}O_{14}H^{+}_{2}$	-0.52	464785	N_3
815.5764	2	14	2	$C_{80}H_{148}N_{20}O_{15}H^{+}_{2}$	-3.10	14310	N_3
865.1176	2	15	2	$C_{85}H_{157}N_{21}O_{16}H^{+}_{2}$	5.16	20855	N_3
744.52362	2	13	1	$C_{73}H_{134}N_{18}O_{14}H^{+}_{2}$	0.00	87511	N_3
794.06071	2	14	1	$C_{78}H_{143}N_{19}O_{15}H^{+}_{2}$	3.63	22546	N_3
843.59346	2	15	1	$C_{83}H_{152}N_{20}O_{16}H^{+}_{2}$	1.69	5125	N_3
429.8145	4	15	4	$C_{84}H_{158}N_{22}O_{15}H^{+}_{4}$	0.69	3585	N_3
454.58124	4	16	4	$C_{89}H_{167}N_{23}O_{16}H^{+}_{4}$	-0.14	31645	N_3
479.34822	4	17	4	$C_{94}H_{176}N_{24}O_{17}H^{+}_{4}$	-0.39	72829	N_3
504.11536	4	18	4	$C_{99}H_{185}N_{25}O_{18}H^{+}_{4}$	-0.30	122375	N_3
528.8822	4	19	4	$C_{104}H_{194}N_{26}O_{19}H^{+}_{4}$	-0.79	191464	N_3
578.41665	4	21	4	$C_{114}H_{212}N_{28}O_{21}H^{+}_{4}$	-0.30	261909	N_3
603.18408	4	22	4	$C_{119}H_{221}N_{29}O_{22}H^{+}_{4}$	0.25	195540	N_3
627.9511	4	23	4	$C_{124}H_{230}N_{30}O_{23}H^{+}_{4}$	0.11	91259	N_3
652.71949	4	24	4	$C_{129}H_{239}N_{31}O_{24}H^{+}_{4}$	2.08	25605	N_3
677.48553	4	25	4	$C_{134}H_{248}N_{32}O_{25}H^{+}_{4}$	0.43	4649	N_3
415.80737	4	13	5	$C_{81}H_{154}N_{22}O_{14}H^{+}_{4}$	-0.67	4552	N_3
440.57485	4	14	5	$C_{86}H_{163}N_{23}O_{15}H^{+}_{4}$	0.22	15139	N_3
490.10873	4	16	5	$C_{96}H_{181}N_{25}O_{17}H^{+}_{4}$	-0.47	133165	N_3
514.8761	4	17	5	$C_{101}H_{190}N_{26}O_{18}H^{+}_{4}$	0.07	144570	N_3
539.64306	4	18	5	$C_{106}H_{199}N_{27}O_{19}H^{+}_{4}$	-0.20	250292	N_3
589.17716	4	20	5	$C_{116}H_{217}N_{29}O_{21}H^{+}_{4}$	-0.36	237417	N_3
613.94419	4	21	5	$C_{121}H_{226}N_{30}O_{22}H^{+}_{4}$	-0.47	67214	N_3
638.71223	4	22	5	$C_{126}H_{235}N_{31}O_{23}H^{+}_{4}$	1.02	112646	N_3
426.56839	4	14	6	$C_{83}H_{159}N_{23}O_{14}H^{+}_{4}$	0.45	4224	N_3
451.33495	4	15	6	$C_{88}H_{168}N_{24}O_{15}H^{+}_{4}$	-0.78	2658	N_3
476.1023	4	16	6	$C_{93}H_{177}N_{25}O_{16}H^{+}_{4}$	-0.22	47074	N_3
500.86909	4	17	6	$C_{98}H_{186}N_{26}O_{17}H^{+}_{4}$	-0.84	84751	N_3
525.63622	4	18	6	$C_{103}H_{195}N_{27}O_{18}H^{+}_{4}$	-0.75	81187	N_3
550.40309	4	19	6	$C_{108}H_{204}N_{28}O_{19}H^{+}_{4}$	-1.14	102463	N_3
575.17132	4	20	6	$C_{113}H_{213}N_{29}O_{20}H^{+}_{4}$	0.87	34897	N_3
599.93735	4	21	6	$C_{118}H_{222}N_{30}O_{21}H^{+}_{4}$	-0.95	14584	N_3
535.71897	3	14	2	$C_{80}H_{149}N_{17}O_{16}H^{+}_{3}$	-1.08	10714	OH
568.74198	3	15	2	$C_{85}H_{158}N_{18}O_{17}H^{+}_{3}$	-0.66	18990	OH
601.76466	3	16	2	$C_{90}H_{167}N_{19}O_{18}H^{+}_{3}$	-0.83	58650	OH
634.78816	3	17	2	$C_{95}H_{176}N_{20}O_{19}H^{+}_{3}$	0.31	40551	OH
667.81176	3	18	2	$C_{100}H_{185}N_{21}O_{20}H^{+}_{3}$	1.48	19999	OH
700.83677	3	19	2	$C_{105}H_{194}N_{22}O_{21}H^{+}_{3}$	4.56	7341	OH
450.99815	3	11	3	$C_{67}H_{127}N_{15}O_{13}H^{+}_{3}$	-0.86	5046	OH
484.022	3	12	3	$C_{72}H_{136}N_{16}O_{14}H^{+}_{3}$	1.36	70547	OH
517.0439	3	13	3	$C_{77}H_{145}N_{17}O_{15}H^{+}_{3}$	-0.47	172407	OH
550.06698	3	14	3	$C_{82}H_{154}N_{18}O_{16}H^{+}_{3}$	0.05	397389	OH
583.08977	3	15	3	$C_{87}H_{163}N_{19}O_{17}H^{+}_{3}$	0.03	504750	OH
616.1124	3	16	3	$C_{92}H_{172}N_{20}O_{18}H^{+}_{3}$	-0.26	357253	OH
649.13607	3	17	3	$C_{97}H_{181}N_{21}O_{19}H^{+}_{3}$	1.09	374768	OH
682.15986	3	18	3	$C_{102}H_{190}N_{22}O_{20}H^{+}_{3}$	2.48	242821	OH

715.18192	3	19	3	$C_{107}H_{199}N_{23}O_{21}H^+_3$	1.32	95735	OH
748.20474	3	20	3	$C_{112}H_{208}N_{24}O_{22}H^+_3$	1.29	21659	OH
399.30042	3	9	4	$C_{59}H_{114}N_{14}O_{11}H^+_3$	0.23	1425	OH
432.32416	3	10	4	$C_{64}H_{123}N_{15}O_{12}H^+_3$	2.38	3653	OH
465.34516	3	11	4	$C_{69}H_{132}N_{16}O_{13}H^+_3$	-1.67	62034	OH
498.36816	3	12	4	$C_{74}H_{141}N_{17}O_{14}H^+_3$	-1.16	136416	OH
531.39141	3	13	4	$C_{79}H_{150}N_{18}O_{15}H^+_3$	-0.25	272054	OH
564.41366	3	14	4	$C_{84}H_{159}N_{19}O_{16}H^+_3$	-1.22	492699	OH
597.43701	3	15	4	$C_{89}H_{168}N_{20}O_{17}H^+_3$	-0.24	514379	OH
630.4601	3	16	4	$C_{94}H_{177}N_{21}O_{18}H^+_3$	0.22	360177	OH
663.48387	3	17	4	$C_{99}H_{186}N_{22}O_{19}H^+_3$	1.67	224944	OH
696.50752	3	18	4	$C_{104}H_{195}N_{23}O_{20}H^+_3$	2.80	35353	OH
413.6475	3	11	5	$C_{61}H_{119}N_{15}O_{11}H^+_3$	-0.55	1874	OH
446.66978	3	12	5	$C_{66}H_{128}N_{16}O_{12}H^+_3$	-1.68	12121	OH
512.71573	3	13	5	$C_{76}H_{146}N_{18}O_{14}H^+_3$	-0.80	120136	OH
545.73829	3	14	5	$C_{81}H_{155}N_{19}O_{15}H^+_3$	-1.20	196729	OH
611.78425	3	16	5	$C_{91}H_{173}N_{21}O_{17}H^+_3$	-0.50	108919	OH
420.31647	4	14	6	$C_{83}H_{160}N_{20}O_{15}H^+_4$	-0.26	5148	OH
445.08358	4	15	6	$C_{88}H_{169}N_{21}O_{16}H^+_4$	-0.23	13283	OH
469.8506	4	16	6	$C_{93}H_{178}N_{22}O_{17}H^+_4$	-0.39	69522	OH
494.61744	4	17	6	$C_{98}H_{187}N_{23}O_{18}H^+_4$	-0.91	103646	OH
519.38455	4	18	6	$C_{103}H_{196}N_{24}O_{19}H^+_4$	-0.85	95344	OH
544.15104	4	19	6	$C_{108}H_{205}N_{25}O_{20}H^+_4$	-1.94	89390	OH
568.91835	4	20	6	$C_{113}H_{214}N_{26}O_{21}H^+_4$	-1.49	56700	OH
593.68722	4	21	6	$C_{118}H_{223}N_{27}O_{22}H^+_4$	1.55	11092	OH
423.56233	4	14	4	$C_{84}H_{159}N_{19}O_{16}H^+_4$	-0.59	1695	OH
448.32907	4	15	4	$C_{89}H_{168}N_{20}O_{17}H^+_4$	-1.37	2571	OH
473.09576	4	16	4	$C_{94}H_{177}N_{21}O_{18}H^+_4$	-2.17	7706	OH
522.63075	4	17	4	$C_{104}H_{195}N_{23}O_{20}H^+_4$	-0.47	122737	OH
547.39739	4	18	4	$C_{109}H_{204}N_{24}O_{21}H^+_4$	-1.30	131969	OH
572.16408	4	19	4	$C_{114}H_{213}N_{25}O_{22}H^+_4$	-1.96	94777	OH
621.69847	4	21	4	$C_{124}H_{231}N_{27}O_{24}H^+_4$	-1.51	24223	OH
646.46779	4	22	4	$C_{129}H_{240}N_{28}O_{25}H^+_4$	1.98	8890	OH
409.5562	4	14	5	$C_{81}H_{155}N_{19}O_{15}H^+_4$	0.42	4316	OH
434.32336	4	15	5	$C_{86}H_{164}N_{20}O_{16}H^+_4$	0.53	14606	OH
459.09009	4	16	5	$C_{91}H_{173}N_{21}O_{17}H^+_4$	-0.32	68971	OH
508.62404	4	18	5	$C_{101}H_{191}N_{23}O_{19}H^+_4$	-0.79	170206	OH
431.07733	4	14	7	$C_{85}H_{165}N_{21}O_{15}H^+_4$	0.47	5904	OH
455.84413	4	15	7	$C_{90}H_{174}N_{22}O_{16}H^+_4$	-0.22	16794	OH
480.6112	4	16	7	$C_{95}H_{183}N_{23}O_{17}H^+_4$	-0.28	27880	OH
505.37743	4	17	7	$C_{100}H_{192}N_{24}O_{18}H^+_4$	-1.99	24137	OH
530.1443	4	18	7	$C_{105}H_{201}N_{25}O_{19}H^+_4$	-2.34	18582	OH
554.91243	4	19	7	$C_{110}H_{210}N_{26}O_{20}H^+_4$	-0.39	18632	OH
579.68164	4	20	7	$C_{115}H_{219}N_{27}O_{21}H^+_4$	3.26	5054	OH
				Absolute Average	1		
				Standard deviation	1.41		
				Sum weighted EI N_3	52911795		

Sum weighted Ox N_3	276953792
Ratio	19.1%
Sum weighted El OH	24003444
Sum weighted Ox OH	96024100
Ratio	25%

Table S 5: ECD assignment of P(Ox_{19}-*co*-EI_1)-N_3 Figure 3A in main text

**m/z**	**Charge**	**Chemical assignment**	**Ox**	**EI**	**Fragment assignment**	**Error (ppm)**
187.1441	1	$C_9H_{18}N_2O_2H^+{}_1$	2	0	a2 0EI	-0.02
286.21252	1	$C_{14}H_{27}N_3O_3H^+{}_1$	3	0	a3 0EI	0.01
385.28094	1	$C_{19}H_{36}N_4O_4H^+{}_1$	4	0	a4 0EI	0.02
484.3493	1	$C_{24}H_{45}N_5O_5H^+{}_1$	5	0	a5 0EI	-0.10
583.41778	1	$C_{29}H_{54}N_6O_6H^+{}_1$	6	0	a6 0EI	0.03
682.48614	1	$C_{34}H_{63}N_7O_7H^+{}_1$	7	0	a7 0EI	-0.05
781.55458	1	$C_{39}H_{72}N_8O_8H^+{}_1$	8	0	a8 0EI	-0.01
880.62312	1	$C_{44}H_{81}N_9O_9H^+{}_1$	9	0	a9 0EI	0.13
979.69232	1	$C_{49}H_{90}N_{10}O_{10}H^+{}_1$	10	0	a10 0EI	0.92
1078.76041	1	$C_{54}H_{99}N_{11}O_{11}H^+{}_1$	11	0	a11 0EI	0.54
1177.82817	1	$C_{59}H_{108}N_{12}O_{12}H^+{}_1$	12	0	a12 0EI	-0.06
1276.8962	1	$C_{64}H_{117}N_{13}O_{13}H^+{}_1$	13	0	a13 0EI	-0.36
1375.96509	1	$C_{69}H_{126}N_{14}O_{14}H^+{}_1$	14	0	a14 0EI	0.01
1475.03446	1	$C_{74}H_{135}N_{15}O_{15}H^+{}_1$	15	0	a15 0EI	0.66
1574.10216	1	$C_{79}H_{144}N_{16}O_{16}H^+{}_1$	16	0	a16 0EI	0.17
1673.16682	1	$C_{84}H_{153}N_{17}O_{17}H^+{}_1$	17	0	a17 0EI	-2.09
1772.24155	1	$C_{89}H_{162}N_{18}O_{18}H^+{}_1$	18	0	a18 0EI	1.59
131.11793	1	$C_6H_{14}N_2O_1H^+{}_1$	1	1	a2 1EI	0.31
230.1863	1	$C_{11}H_{23}N_3O_2H^+{}_1$	2	1	a3 1EI	-0.02
329.2547	1	$C_{16}H_{32}N_4O_3H^+{}_1$	3	1	a4 1EI	-0.05
428.3231	1	$C_{21}H_{41}N_5O_4H^+{}_1$	4	1	a5 1EI	-0.07
527.39163	1	$C_{26}H_{50}N_6O_5H^+{}_1$	5	1	a6 1EI	0.16
626.46012	1	$C_{31}H_{59}N_7O_6H^+{}_1$	6	1	a7 1EI	0.26
725.5283	1	$C_{36}H_{68}N_8O_7H^+{}_1$	7	1	a8 1EI	-0.10
824.59696	1	$C_{41}H_{77}N_9O_8H^+{}_1$	8	1	a9 1EI	0.21
923.66497	1	$C_{46}H_{86}N_{10}O_9H^+{}_1$	9	1	a10 1EI	-0.25
1022.73335	1	$C_{51}H_{95}N_{11}O_{10}H^+{}_1$	10	1	a11 1EI	-0.26
1121.80204	1	$C_{56}H_{104}N_{12}O_{11}H^+{}_1$	11	1	a12 1EI	0.01
1220.87059	1	$C_{61}H_{113}N_{13}O_{12}H^+{}_1$	12	1	a13 1EI	0.12
1319.9391	1	$C_{66}H_{122}N_{14}O_{13}H^+{}_1$	13	1	a14 1EI	0.18
1419.00634	1	$C_{71}H_{131}N_{15}O_{14}H^+{}_1$	14	1	a15 1EI	-0.66
1518.07587	1	$C_{76}H_{140}N_{16}O_{15}H^+{}_1$	15	1	a16 1EI	0.12
1617.14498	1	$C_{81}H_{149}N_{17}O_{16}H^+{}_1$	16	1	a17 1EI	0.55
1716.21369	1	$C_{86}H_{158}N_{18}O_{17}H^+{}_1$	17	1	a18 1EI	0.69
1815.28096	1	$C_{91}H_{167}N_{19}O_{18}H^+{}_1$	18	1	a19 1EI	0.02
242.16114	1	$C_{10}H_{19}N_5O_2H^+{}_1$	2	0	x2 0EI	-0.05
341.2296	1	$C_{15}H_{28}N_6O_3H^+{}_1$	3	0	x3 0EI	0.10
440.29797	1	$C_{20}H_{37}N_7O_4H^+{}_1$	4	0	x4 0EI	-0.02
539.36636	1	$C_{25}H_{46}N_8O_5H^+{}_1$	5	0	x5 0EI	-0.06
638.4349	1	$C_{30}H_{55}N_9O_6H^+{}_1$	6	0	x6 0EI	0.15
737.50353	1	$C_{35}H_{64}N_{10}O_7H^+{}_1$	7	0	x7 0EI	0.42
935.63838	1	$C_{45}H_{82}N_{12}O_9H^+{}_1$	9	0	x9 0EI	-1.78
1034.70804	1	$C_{50}H_{91}N_{13}O_{10}H^+{}_1$	10	0	x10 0EI	-0.41
1133.77653	1	$C_{55}H_{100}N_{14}O_{11}H^+{}_1$	11	0	x11 0EI	-0.31

1232.84499	1	$C_{60}H_{109}N_{15}O_{12}H^{+}{}_{1}$	12	0	x12 0EI	-0.24
1331.91245	1	$C_{65}H_{118}N_{16}O_{13}H^{+}{}_{1}$	13	0	x13 0EI	-0.94
836.5715	2	$C_{40}H_{73}N_{11}O_{8}H^{+}{}_{1}$	8	0	x8 0EI	-0.16
285.20341	1	$C_{12}H_{24}N_{6}O_{2}H^{+}{}_{1}$	2	1	x3 1EI	0.21
384.27165	1	$C_{17}H_{33}N_{7}O_{3}H^{+}{}_{1}$	3	1	x4 1EI	-0.30
483.34007	1	$C_{22}H_{42}N_{8}O_{4}H^{+}{}_{1}$	4	1	x5 1EI	-0.22
582.40847	1	$C_{27}H_{51}N_{9}O_{5}H^{+}{}_{1}$	5	1	x6 1EI	-0.21
681.47714	1	$C_{32}H_{60}N_{10}O_{6}H^{+}{}_{1}$	6	1	x7 1EI	0.20
780.54554	1	$C_{37}H_{69}N_{11}O_{7}H^{+}{}_{1}$	7	1	x8 1EI	0.15
879.61387	1	$C_{42}H_{78}N_{12}O_{8}H^{+}{}_{1}$	8	1	x9 1EI	0.04
1077.75097	1	$C_{52}H_{96}N_{14}O_{10}H^{+}{}_{1}$	10	1	x11 1EI	0.29
1176.81899	1	$C_{57}H_{105}N_{15}O_{11}H^{+}{}_{1}$	11	1	x12 1EI	-0.07
1275.88623	1	$C_{62}H_{114}N_{16}O_{12}H^{+}{}_{1}$	12	1	x13 1EI	-0.99
1374.95599	1	$C_{67}H_{123}N_{17}O_{13}H^{+}{}_{1}$	13	1	x14 1EI	0.06
1474.02457	1	$C_{72}H_{132}N_{18}O_{14}H^{+}{}_{1}$	14	1	x15 1EI	0.17
1573.09323	1	$C_{77}H_{141}N_{19}O_{15}H^{+}{}_{1}$	15	1	x16 1EI	0.32
1672.16098	1	$C_{82}H_{150}N_{20}O_{16}H^{+}{}_{1}$	16	1	x17 1EI	-0.10
1771.23076	1	$C_{87}H_{159}N_{21}O_{17}H^{+}{}_{1}$	17	1	x18 1EI	0.68
710.00737	2	$C_{71}H_{131}N_{15}O_{14}H^{+}{}_{2}$	14	1	a15 1EI	0.14
759.54101	2	$C_{76}H_{140}N_{16}O_{15}H^{+}{}_{2}$	15	1	a16 1EI	-0.62
809.07559	2	$C_{81}H_{149}N_{17}O_{16}H^{+}{}_{2}$	16	1	a17 1EI	-0.12
858.61006	2	$C_{86}H_{158}N_{18}O_{17}H^{+}{}_{2}$	17	1	a18 1EI	0.19
908.14461	2	$C_{91}H_{167}N_{19}O_{18}H^{+}{}_{2}$	18	1	a19 1EI	0.56
957.6784	2	$C_{96}H_{176}N_{20}O_{19}H^{+}{}_{2}$	19	1	a20 1EI	0.10
787.05132	2	$C_{77}H_{141}N_{19}O_{15}H^{+}{}_{2}$	0	0	x17 1EI	1.67
836.58383	2	$C_{82}H_{150}N_{20}O_{16}H^{+}{}_{2}$	0	0	x18 1EI	-0.46
886.1183	2	$C_{87}H_{159}N_{21}O_{17}H^{+}{}_{2}$	0	0	x19 1EI	-0.13
935.65262	2	$C_{92}H_{168}N_{22}O_{18}H^{+}{}_{2}$	0	0	x20 1EI	-0.01
964.18223	2	$C_{95}H_{175}N_{23}O_{18}H^{+}{}_{2}$	0	0	CRS-C3H4O	0.71
978.17886	2	$C_{96}H_{175}N_{23}O_{19}H^{+}{}_{2}$	0	0	CRS-C2H4	-0.15
983.69313	2	$C_{98}H_{178}N_{23}O_{18}H^{+}{}_{2}$	0	0	CRS-H2O	-0.16
992.19454	2	$C_{98}H_{179}N_{23}O_{19}H^{+}{}_{2}$	0	0	CRS-H	-0.12
661.79936	3	$C_{98}H_{179}N_{23}O_{19}H^{+}{}_{3}$	0	0	Precursor	0.75
					Average	0.0
					Absolute average	0.31
					Std dev	0.51

Figure S 3: *x*-series fragmentation diagram P(Ox_{19}-*co*-EI_1)-N_3 reverse of Figure 3C in text

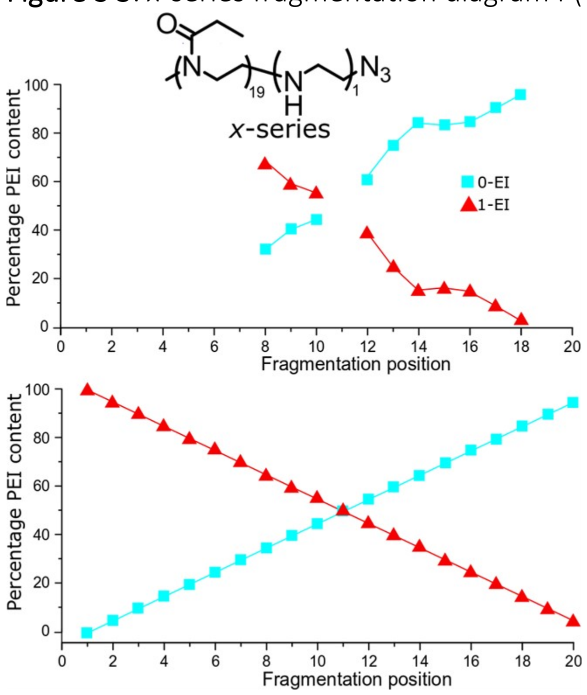

Table S 6: ECD assignment of P(Ox_{19}-*co*-EI_1)-OH Figure 4A

m/z	Charge	Chemical assignment	Pox	EI	Fragment assignment	Error
1845.28943	1	$C_{92}H_{169}N_{19}O_{19}H^+{}_1$	18	1	x19 1EI	-1.12
1746.21835	1	$C_{87}H_{160}N_{18}O_{18}H^+{}_1$	17	1	x18 1EI	-2.71
1647.15545	1	$C_{82}H_{151}N_{17}O_{17}H^+{}_1$	16	1	x17 1EI	0.48
1548.08782	1	$C_{77}H_{142}N_{16}O_{16}H^+{}_1$	15	1	x16 1EI	1.01
1449.0189	1	$C_{72}H_{133}N_{15}O_{15}H^+{}_1$	14	1	x15 1EI	0.73
1349.94865	1	$C_{67}H_{124}N_{14}O_{14}H^+{}_1$	13	1	x14 1EI	-0.57
1250.88033	1	$C_{62}H_{115}N_{13}O_{13}H^+{}_1$	12	1	x13 1EI	-0.54
1151.81232	1	$C_{57}H_{106}N_{12}O_{12}H^+{}_1$	11	1	x12 1EI	-0.24
1052.74394	1	$C_{52}H_{97}N_{11}O_{11}H^+{}_1$	10	1	x11 1EI	-0.23
953.67548	1	$C_{47}H_{88}N_{10}O_{10}H^+{}_1$	9	1	x10 1EI	-0.30
854.60759	1	$C_{42}H_{79}N_9O_9H^+{}_1$	8	1	x9 1EI	0.28
755.53899	1	$C_{37}H_{70}N_8O_8H^+{}_1$	7	1	x8 1EI	0.07
656.47038	1	$C_{32}H_{61}N_7O_7H^+{}_1$	6	1	x7 1EI	-0.22
557.40212	1	$C_{27}H_{52}N_6O_6H^+{}_1$	5	1	x6 1EI	0.02
458.3337	1	$C_{22}H_{43}N_5O_5H^+{}_1$	4	1	x5 1EI	0.01
359.26527	1	$C_{17}H_{34}N_4O_4H^+{}_1$	3	1	x4 1EI	-0.03
260.19684	1	$C_{12}H_{25}N_3O_3H^+{}_1$	2	1	x3 1EI	-0.11
161.12848	1	$C_7H_{16}N_2O_2H^+{}_1$	1	1	x2 1EI	0.16
1772.24212	1	$C_{89}H_{162}N_{18}O_{18}H^+{}_1$	18	0	a18	1.91
1673.17031	1	$C_{84}H_{153}N_{17}O_{17}H^+{}_1$	17	0	a17	0.00
1574.1017	1	$C_{79}H_{144}N_{16}O_{16}H^+{}_1$	16	0	a16	-0.13
1475.03426	1	$C_{74}H_{135}N_{15}O_{15}H^+{}_1$	15	0	a15	0.53
1375.96575	1	$C_{69}H_{126}N_{14}O_{14}H^+{}_1$	14	0	a14	0.49
1276.89587	1	$C_{64}H_{117}N_{13}O_{13}H^+{}_1$	13	0	a13	-0.62
1177.82882	1	$C_{59}H_{108}N_{12}O_{12}H^+{}_1$	12	0	a12	0.49
1078.76026	1	$C_{54}H_{99}N_{11}O_{11}H^+{}_1$	11	0	a11	0.40
880.62304	1	$C_{44}H_{81}N_9O_9H^+{}_1$	9	0	a9	0.04
781.55459	1	$C_{39}H_{72}N_8O_8H^+{}_1$	8	0	a8	0.00
682.48622	1	$C_{34}H_{63}N_7O_7H^+{}_1$	7	0	a7	0.07
583.4178	1	$C_{29}H_{54}N_6O_6H^+{}_1$	6	0	a6	0.07
484.34935	1	$C_{24}H_{45}N_5O_5H^+{}_1$	5	0	a5	0.01
385.28093	1	$C_{19}H_{36}N_4O_4H^+{}_1$	4	0	a4	-0.01
286.21249	1	$C_{14}H_{27}N_3O_3H^+{}_1$	3	0	a3	-0.10
187.1441	1	$C_9H_{18}N_2O_2H^+{}_1$	2	0	a2	-0.02
1716.21872	1	$C_{86}H_{158}N_{18}O_{17}H^+{}_1$	17	1	a18 1EI	3.62
1617.14977	1	$C_{81}H_{149}N_{17}O_{16}H^+{}_1$	16	1	a17 1EI	3.51
1518.07829	1	$C_{76}H_{140}N_{16}O_{15}H^+{}_1$	15	1	a16 1EI	1.72
1419.00635	1	$C_{71}H_{131}N_{15}O_{14}H^+{}_1$	14	1	a15 1EI	-0.65
1319.9405	1	$C_{66}H_{122}N_{14}O_{13}H^+{}_1$	13	1	a14 1EI	1.25
1220.87048	1	$C_{61}H_{113}N_{13}O_{12}H^+{}_1$	12	1	a13 1EI	0.03
1121.80202	1	$C_{56}H_{104}N_{12}O_{11}H^+{}_1$	11	1	a12 1EI	-0.01
1022.7329	1	$C_{51}H_{95}N_{11}O_{10}H^+{}_1$	10	1	a11 1EI	-0.70
824.59673	1	$C_{41}H_{77}N_9O_8H^+{}_1$	8	1	a9 1EI	-0.07
725.52816	1	$C_{36}H_{68}N_8O_7H^+{}_1$	7	1	a8 1EI	-0.29
626.46009	1	$C_{31}H_{59}N_7O_6H^+{}_1$	6	1	a7 1EI	0.21

527.39158	1	$C_{26}H_{50}N_6O_5H^+_1$	5	1	a6 1EI	0.07
428.32312	1	$C_{21}H_{41}N_5O_4H^+_1$	4	1	a5 1EI	-0.03
329.25467	1	$C_{16}H_{32}N_4O_3H^+_1$	3	1	a4 1EI	-0.14
230.1863	1	$C_{11}H_{23}N_3O_2H^+_1$	2	1	a3 1EI	-0.02
131.11794	1	$C_6H_{14}N_2O_1H^+_1$	1	1	a2 1EI	0.38
908.14387	2	$C_{91}H_{167}N_{19}O_{18}H^+_2$	0	0	a19 1EI	-0.25
873.61482	2	$C_{87}H_{160}N_{18}O_{18}H^+_2$	0	0	x18 1EI	-0.41
					Absolute Average	0.55
					Std dev	0.95

Figure S 4: Fragmentation of p(22EI-2EI)OH

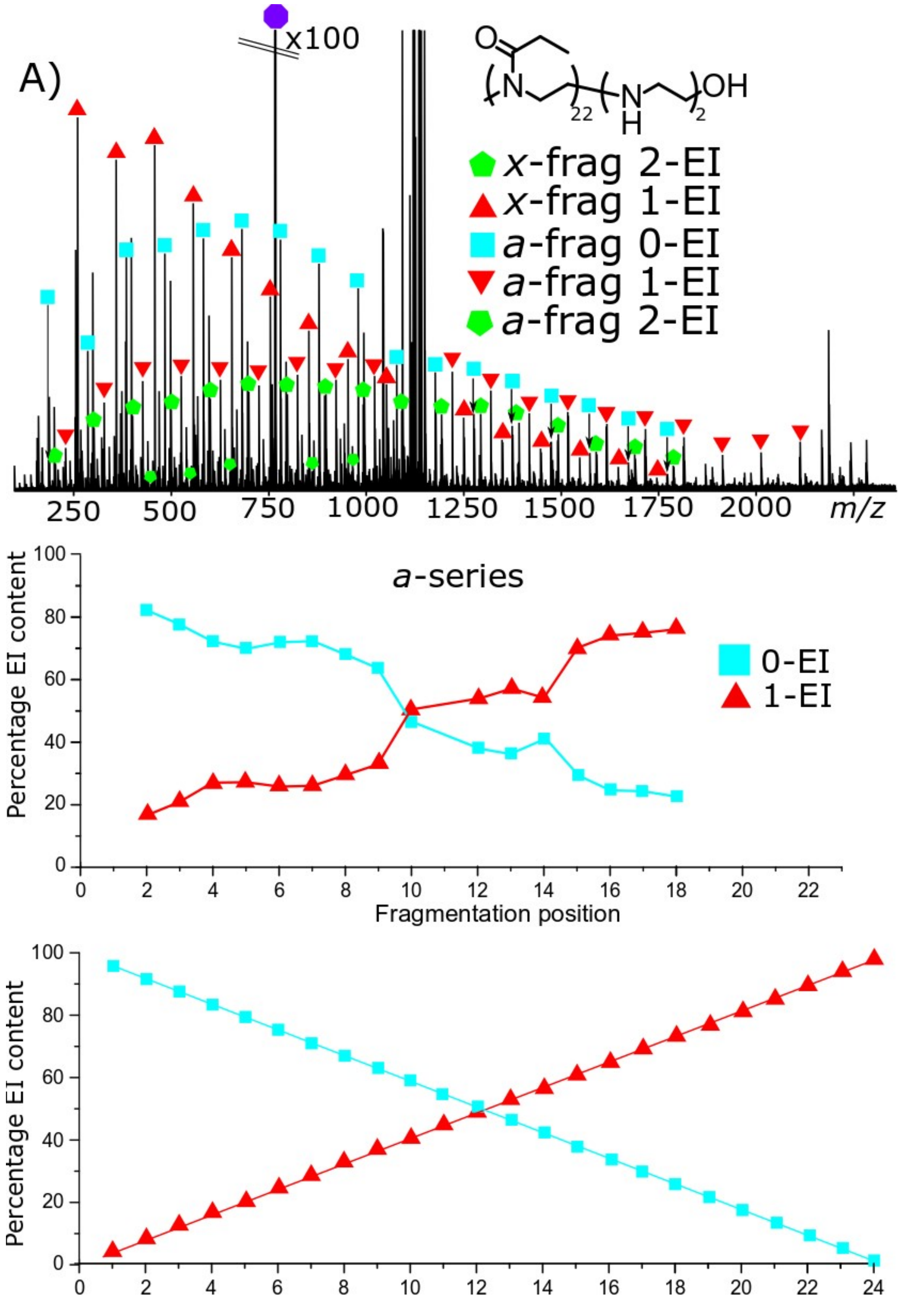

Table S 7: ECD fragmentation assignment of a p(22Pox-*r*-2EI)OH Figure 5 in main text

m/z	Chemical assignment	Pox	EI	Fragment assignment	Error
1475.03221	$C_{74}H_{135}N_{15}O_{15}H^{+}{}_{1}$	15	0	a15 0EI	-0.86
1375.96378	$C_{69}H_{126}N_{14}O_{14}H^{+}{}_{1}$	14	0	a14 0EI	-0.94
1276.89628	$C_{64}H_{117}N_{13}O_{13}H^{+}{}_{1}$	13	0	a13 0EI	-0.30
1177.82835	$C_{59}H_{108}N_{12}O_{12}H^{+}{}_{1}$	12	0	a12 0EI	0.09
1078.75978	$C_{54}H_{99}N_{11}O_{11}H^{+}{}_{1}$	11	0	a11 0EI	-0.05
979.69139	$C_{49}H_{90}N_{10}O_{10}H^{+}{}_{1}$	10	0	a10 0EI	-0.03
880.62234	$C_{44}H_{81}N_{9}O_{9}H^{+}{}_{1}$	9	0	a9 0EI	-0.75
781.5544	$C_{39}H_{72}N_{8}O_{8}H^{+}{}_{1}$	8	0	a8 0EI	-0.24
682.48626	$C_{34}H_{63}N_{7}O_{7}H^{+}{}_{1}$	7	0	a7 0EI	0.13
583.41778	$C_{29}H_{54}N_{6}O_{6}H^{+}{}_{1}$	6	0	a6 0EI	0.03
484.34937	$C_{24}H_{45}N_{5}O_{5}H^{+}{}_{1}$	5	0	a5 0EI	0.05
385.28093	$C_{19}H_{36}N_{4}O_{4}H^{+}{}_{1}$	4	0	a4 0EI	-0.01
286.21254	$C_{14}H_{27}N_{3}O_{3}H^{+}{}_{1}$	3	0	a3 0EI	0.08
187.1441	$C_{9}H_{18}N_{2}O_{2}H^{+}{}_{1}$	2	0	a2 0EI	-0.02
1815.27957	$C_{91}H_{167}N_{19}O_{18}H^{+}{}_{1}$	18	1	a19 1EI	-0.75
1716.21064	$C_{86}H_{158}N_{18}O_{17}H^{+}{}_{1}$	17	1	a18 1EI	-1.09
1617.14335	$C_{81}H_{149}N_{17}O_{16}H^{+}{}_{1}$	16	1	a17 1EI	-0.46
1518.07535	$C_{76}H_{140}N_{16}O_{15}H^{+}{}_{1}$	15	1	a16 1EI	-0.22
1419.00654	$C_{71}H_{131}N_{15}O_{14}H^{+}{}_{1}$	14	1	a15 1EI	-0.51
1319.93867	$C_{66}H_{122}N_{14}O_{13}H^{+}{}_{1}$	13	1	a14 1EI	-0.14
1220.87012	$C_{61}H_{113}N_{13}O_{12}H^{+}{}_{1}$	12	1	a13 1EI	-0.26
1121.80148	$C_{56}H_{104}N_{12}O_{11}H^{+}{}_{1}$	11	1	a12 1EI	-0.49
1022.73343	$C_{51}H_{95}N_{11}O_{10}H^{+}{}_{1}$	10	1	a11 1EI	-0.18
923.66568	$C_{46}H_{86}N_{10}O_{9}H^{+}{}_{1}$	9	1	a10 1EI	0.52
824.59709	$C_{41}H_{77}N_{9}O_{8}H^{+}{}_{1}$	8	1	a9 1EI	0.37
725.52848	$C_{36}H_{68}N_{8}O_{7}H^{+}{}_{1}$	7	1	a8 1EI	0.15
626.45991	$C_{31}H_{59}N_{7}O_{6}H^{+}{}_{1}$	6	1	a7 1EI	-0.08
527.39164	$C_{26}H_{50}N_{6}O_{5}H^{+}{}_{1}$	5	1	a6 1EI	0.18
428.32315	$C_{21}H_{41}N_{5}O_{4}H^{+}{}_{1}$	4	1	a5 1EI	0.04
329.2547	$C_{16}H_{32}N_{4}O_{3}H^{+}{}_{1}$	3	1	a4 1EI	-0.05
230.18631	$C_{11}H_{23}N_{3}O_{2}H^{+}{}_{1}$	2	1	a3 1EI	0.03
131.1179	$C_{6}H_{14}N_{2}O_{1}H^{+}{}_{1}$	1	1	a2 1EI	0.08
1660.18466	$C_{83}H_{154}N_{18}O_{16}H^{+}{}_{1}$	16	2	a18 2EI	-0.99
1561.11916	$C_{78}H_{145}N_{17}O_{15}H^{+}{}_{1}$	15	2	a17 2EI	0.82
1462.04825	$C_{73}H_{136}N_{16}O_{14}H^{+}{}_{1}$	14	2	a16 2EI	-0.83
1362.97958	$C_{68}H_{127}N_{15}O_{13}H^{+}{}_{1}$	13	2	a15 2EI	-1.08
1263.91302	$C_{63}H_{118}N_{14}O_{12}H^{+}{}_{1}$	12	2	a14 2EI	0.30
1164.84385	$C_{58}H_{109}N_{13}O_{11}H^{+}{}_{1}$	11	2	a13 2EI	-0.32
1065.77556	$C_{53}H_{100}N_{12}O_{10}H^{+}{}_{1}$	10	2	a12 2EI	-0.24
966.70717	$C_{48}H_{91}N_{11}O_{9}H^{+}{}_{1}$	9	2	a11 2EI	-0.24
867.63848	$C_{43}H_{82}N_{10}O_{8}H^{+}{}_{1}$	8	2	a10 2EI	-0.58
768.56996	$C_{38}H_{73}N_{9}O_{7}H^{+}{}_{1}$	7	2	a9 2EI	-0.80
669.50388	$C_{33}H_{64}N_{8}O_{6}H^{+}{}_{1}$	6	2	a8 2EI	2.57
570.43371	$C_{28}H_{55}N_{7}O_{5}H^{+}{}_{1}$	5	2	a7 2EI	-0.06
471.36517	$C_{23}H_{46}N_{6}O_{4}H^{+}{}_{1}$	4	2	a6 2EI	-0.34

372.29678	$C_{18}H_{37}N_5O_3H^+_1$	3	2	a5 2EI	-0.37
836.57157	$C_{40}H_{73}N_{11}O_8H^+_1$	8	0	x8 0EI	-0.08
737.50261	$C_{35}H_{64}N_{10}O_7H^+_1$	7	0	x7 0EI	-0.83
638.43411	$C_{30}H_{55}N_9O_6H^+_1$	6	0	x6 0EI	-1.09
539.36655	$C_{25}H_{46}N_8O_5H^+_1$	5	0	x5 0EI	0.29
440.29788	$C_{20}H_{37}N_7O_4H^+_1$	4	0	x4 0EI	-0.23
341.22963	$C_{15}H_{28}N_6O_3H^+_1$	3	0	x3 0EI	0.19
1176.81829	$C_{57}H_{105}N_{15}O_{11}H^+_1$	11	1	x12 1EI	-0.67
1077.75143	$C_{52}H_{96}N_{14}O_{10}H^+_1$	10	1	x11 1EI	0.71
978.68234	$C_{47}H_{87}N_{13}O_9H^+_1$	9	1	x10 1EI	0.09
879.61465	$C_{42}H_{78}N_{12}O_8H^+_1$	8	1	x9 1EI	0.93
780.54511	$C_{37}H_{69}N_{11}O_7H^+_1$	7	1	x8 1EI	-0.40
681.47731	$C_{32}H_{60}N_{10}O_6H^+_1$	6	1	x7 1EI	0.45
582.40861	$C_{27}H_{51}N_9O_5H^+_1$	5	1	x6 1EI	0.03
483.34008	$C_{22}H_{42}N_8O_4H^+_1$	4	1	x5 1EI	-0.20
384.2717	$C_{17}H_{33}N_7O_3H^+_1$	3	1	x4 1EI	-0.17
285.20334	$C_{12}H_{24}N_6O_2H^+_1$	2	1	x3 1EI	-0.04
643.12371	$C_{95}H_{175}N_{23}O_{18}H^+_3$	0	0	Precursor	0.39
964.18115	$C_{95}H_{175}N_{23}O_{18}H^+_2$	0	0	CRS-H	-0.41
929.66506	$C_{93}H_{172}N_{20}O_{18}H^+_2$	18	0	a20 2EI	-0.15
880.13063	$C_{88}H_{163}N_{19}O_{17}H^+_2$	0	0	a19 2EI	-0.41
830.59674	$C_{83}H_{154}N_{18}O_{16}H^+_2$	0	0	a18 2EI	-0.06
781.06314	$C_{78}H_{145}N_{17}O_{15}H^+_2$	0	0	a17 2EI	0.72
731.52778	$C_{73}H_{136}N_{16}O_{14}H^+_2$	0	0	a16 2EI	-0.81
				Average	0.65
				Standard Deviation	1

Table S 8: ECD assignment of P(Ox_{17}-*co*-EI_4)-OH Figure 5A

m/z	Charge	Chemical assignment	Pox	EI	Fragment assignment	Error
854.60736	1	$C_{42}H_{79}N_9O_9H^+_1$	7	1	x8 1EI	0.01
755.53934	1	$C_{37}H_{70}N_8O_8H^+_1$	6	1	x7 1EI	0.53
656.47027	1	$C_{32}H_{61}N_7O_7H^+_1$	5	1	x6 1EI	-0.39
557.40207	1	$C_{27}H_{52}N_6O_6H^+_1$	4	1	x5 1EI	-0.07
359.26525	1	$C_{17}H_{34}N_4O_4H^+_1$	3	1	x4 1EI	-0.09
260.19687	1	$C_{12}H_{25}N_3O_3H^+_1$	2	1	x3 1EI	0.01
161.12847	1	$C_7H_{16}N_2O_2H^+_1$	1	1	x2 1EI	0.10
1194.8543	1	$C_{59}H_{111}N_{13}O_{12}H^+_1$	11	2	x13 2EI	-0.41
1095.78631	1	$C_{54}H_{102}N_{12}O_{11}H^+_1$	10	2	x12 2EI	-0.06
996.71797	1	$C_{49}H_{93}N_{11}O_{10}H^+_1$	9	2	x11 2EI	0.01
897.64943	1	$C_{44}H_{84}N_{10}O_9H^+_1$	8	2	x10 2EI	-0.13
798.58125	1	$C_{39}H_{75}N_9O_8H^+_1$	7	2	x9 2EI	0.14
699.51273	1	$C_{34}H_{66}N_8O_7H^+_1$	6	2	x8 2EI	0.01
600.44422	1	$C_{29}H_{57}N_7O_6H^+_1$	5	2	x7 2EI	-0.15
501.37589	1	$C_{24}H_{48}N_6O_5H^+_1$	4	2	x6 2EI	-0.01
402.3075	1	$C_{19}H_{39}N_5O_4H^+_1$	3	2	x5 2EI	0.05
303.23908	1	$C_{14}H_{30}N_4O_3H^+_1$	2	2	x4 2EI	0.04
204.17065	1	$C_9H_{21}N_3O_2H^+_1$	1	2	x3 2EI	-0.02
105.10229	1	$C_4H_{12}N_2O_1H^+_1$	0	2	x2 2EI	0.48
1535.10141	1	$C_{76}H_{143}N_{17}O_{15}H^+_1$	14	3	x18 3EI	-0.54
1436.03455	1	$C_{71}H_{134}N_{16}O_{14}H^+_1$	13	3	x17 3EI	0.51
1336.96507	1	$C_{66}H_{125}N_{15}O_{13}H^+_1$	12	3	x16 3EI	-0.25
1237.89601	1	$C_{61}H_{116}N_{14}O_{12}H^+_1$	11	3	x15 3EI	-0.79
1138.82879	1	$C_{56}H_{107}N_{13}O_{11}H^+_1$	10	3	x14 3EI	0.19
1039.75999	1	$C_{51}H_{98}N_{12}O_{10}H^+_1$	9	3	x13 3EI	-0.17
940.69162	1	$C_{46}H_{89}N_{11}O_9H^+_1$	8	3	x12 3EI	-0.14
841.6234	1	$C_{41}H_{80}N_{10}O_8H^+_1$	7	3	x11 3EI	0.08
742.55497	1	$C_{36}H_{71}N_9O_7H^+_1$	6	3	x10 3EI	0.06
643.48647	1	$C_{31}H_{62}N_8O_6H^+_1$	5	3	x9 3EI	-0.06
544.41808	1	$C_{26}H_{53}N_7O_5H^+_1$	4	3	x8 3EI	-0.03
445.34971	1	$C_{21}H_{44}N_6O_4H^+_1$	3	3	x7 3EI	0.07
346.28129	1	$C_{16}H_{35}N_5O_3H^+_1$	2	3	x6 3EI	0.07
247.21285	1	$C_{11}H_{26}N_4O_2H^+_1$	1	3	x5 3EI	-0.01
148.14447	1	$C_6H_{17}N_3O_1H^+_1$	0	3	x4 3EI	0.21
1380.00773	1	$C_{68}H_{130}N_{16}O_{13}H^+_1$	11	4	x16 4EI	0.09
1280.94012	1	$C_{63}H_{121}N_{15}O_{12}H^+_1$	10	4	x15 4EI	0.73
1082.80326	1	$C_{53}H_{103}N_{13}O_{10}H^+_1$	9	4	x14 4EI	0.83
983.73418	1	$C_{48}H_{94}N_{12}O_9H^+_1$	8	4	x13 4EI	0.23
884.66587	1	$C_{43}H_{85}N_{11}O_8H^+_1$	7	4	x12 4EI	0.38
785.5971	1	$C_{38}H_{76}N_{10}O_7H^+_1$	6	4	x11 4EI	-0.03
686.52842	1	$C_{33}H_{67}N_9O_6H^+_1$	5	4	x10 4EI	-0.42
587.46061	1	$C_{28}H_{58}N_8O_5H^+_1$	4	4	x9 4EI	0.54
488.39204	1	$C_{23}H_{49}N_7O_4H^+_1$	3	4	x8 4EI	0.33
389.32349	1	$C_{18}H_{40}N_6O_3H^+_1$	2	4	x7 4EI	0.06
290.25501	1	$C_{13}H_{31}N_5O_2H^+_1$	1	4	x6 4EI	-0.14
191.18667	1	$C_8H_{22}N_4O_1H^+_1$	0	4	x5 4EI	0.17

1177.82781	1	$C_{59}H_{108}N_{12}O_{12}H^{+}_{1}$	11	0	a11	-0.37
1078.75988	1	$C_{54}H_{99}N_{11}O_{11}H^{+}_{1}$	10	0	a10	0.05
979.69141	1	$C_{49}H_{90}N_{10}O_{10}H^{+}_{1}$	9	0	a9	-0.01
880.62278	1	$C_{44}H_{81}N_{9}O_{9}H^{+}_{1}$	8	0	a8	-0.25
682.48614	1	$C_{34}H_{63}N_{7}O_{7}H^{+}_{1}$	7	0	a7	-0.05
583.41781	1	$C_{29}H_{54}N_{6}O_{6}H^{+}_{1}$	6	0	a6	0.09
484.34937	1	$C_{24}H_{45}N_{5}O_{5}H^{+}_{1}$	5	0	a5	0.05
385.28096	1	$C_{19}H_{36}N_{4}O_{4}H^{+}_{1}$	4	0	a4	0.07
286.21252	1	$C_{14}H_{27}N_{3}O_{3}H^{+}_{1}$	3	0	a3	0.01
187.1441	1	$C_{9}H_{18}N_{2}O_{2}H^{+}_{1}$	2	0	a2	-0.02
1518.07434	1	$C_{76}H_{140}N_{16}O_{15}H^{+}_{1}$	15	1	a16 1EI	-0.89
1419.00646	1	$C_{71}H_{131}N_{15}O_{14}H^{+}_{1}$	14	1	a15 1EI	-0.57
1319.93756	1	$C_{66}H_{122}N_{14}O_{13}H^{+}_{1}$	13	1	a14 1EI	-0.98
1220.8705	1	$C_{61}H_{113}N_{13}O_{12}H^{+}_{1}$	12	1	a13 1EI	0.05
1121.80215	1	$C_{56}H_{104}N_{12}O_{11}H^{+}_{1}$	11	1	a12 1EI	0.11
1022.7336	1	$C_{51}H_{95}N_{11}O_{10}H^{+}_{1}$	10	1	a11 1EI	-0.01
923.6649	1	$C_{46}H_{86}N_{10}O_{9}H^{+}_{1}$	9	1	a10 1EI	-0.33
824.5965	1	$C_{41}H_{77}N_{9}O_{8}H^{+}_{1}$	8	1	a9 1EI	-0.35
725.52837	1	$C_{36}H_{68}N_{8}O_{7}H^{+}_{1}$	7	1	a8 1EI	0.00
626.46009	1	$C_{31}H_{59}N_{7}O_{6}H^{+}_{1}$	6	1	a7 1EI	0.21
527.39156	1	$C_{26}H_{50}N_{6}O_{5}H^{+}_{1}$	5	1	a6 1EI	0.03
428.32314	1	$C_{21}H_{41}N_{5}O_{4}H^{+}_{1}$	4	1	a5 1EI	0.02
329.25473	1	$C_{16}H_{32}N_{4}O_{3}H^{+}_{1}$	3	1	a4 1EI	0.04
230.1863	1	$C_{11}H_{23}N_{3}O_{2}H^{+}_{1}$	2	1	a3 1EI	-0.02
131.11792	1	$C_{6}H_{14}N_{2}O_{1}H^{+}_{1}$	1	1	a2 1EI	0.23
1660.1817	1	$C_{83}H_{154}N_{18}O_{16}H^{+}_{1}$	16	2	a18 2EI	-2.77
1561.11682	1	$C_{78}H_{145}N_{17}O_{15}H^{+}_{1}$	15	2	a17 2EI	-0.68
1462.04918	1	$C_{73}H_{136}N_{16}O_{14}H^{+}_{1}$	14	2	a16 2EI	-0.20
1362.98083	1	$C_{68}H_{127}N_{15}O_{13}H^{+}_{1}$	13	2	a15 2EI	-0.17
1263.91245	1	$C_{63}H_{118}N_{14}O_{12}H^{+}_{1}$	12	2	a14 2EI	-0.15
1164.84406	1	$C_{58}H_{109}N_{13}O_{11}H^{+}_{1}$	11	2	a13 2EI	-0.14
1065.77591	1	$C_{53}H_{100}N_{12}O_{10}H^{+}_{1}$	10	2	a12 2EI	0.09
966.70755	1	$C_{48}H_{91}N_{11}O_{9}H^{+}_{1}$	9	2	a11 2EI	0.16
867.63895	1	$C_{43}H_{82}N_{10}O_{8}H^{+}_{1}$	8	2	a10 2EI	-0.04
768.57064	1	$C_{38}H_{73}N_{9}O_{7}H^{+}_{1}$	7	2	a9 2EI	0.09
669.50216	1	$C_{33}H_{64}N_{8}O_{6}H^{+}_{1}$	6	2	a8 2EI	0.00
570.43366	1	$C_{28}H_{55}N_{7}O_{5}H^{+}_{1}$	5	2	a7 2EI	-0.15
471.36529	1	$C_{23}H_{46}N_{6}O_{4}H^{+}_{1}$	4	2	a6 2EI	-0.09
372.29689	1	$C_{18}H_{37}N_{5}O_{3}H^{+}_{1}$	3	2	a5 2EI	-0.07
273.22849	1	$C_{13}H_{28}N_{4}O_{2}H^{+}_{1}$	2	2	a4 2EI	-0.05
174.16009	1	$C_{8}H_{19}N_{3}O_{1}H^{+}_{1}$	1	2	a3 2EI	0.01
1604.15787	1	$C_{80}H_{150}N_{18}O_{15}H^{+}_{1}$	15	3	a17 3EI	-1.38
1505.09109	1	$C_{75}H_{141}N_{17}O_{14}H^{+}_{1}$	14	3	a16 3EI	-0.38
1406.02359	1	$C_{70}H_{132}N_{16}O_{13}H^{+}_{1}$	13	3	a15 3EI	0.24
1306.95465	1	$C_{65}H_{123}N_{15}O_{12}H^{+}_{1}$	12	3	a14 3EI	-0.15
1207.88792	1	$C_{60}H_{114}N_{14}O_{11}H^{+}_{1}$	11	3	a13 3EI	1.24
1108.81863	1	$C_{55}H_{105}N_{13}O_{10}H^{+}_{1}$	10	3	a12 3EI	0.56
1009.7498	1	$C_{50}H_{96}N_{12}O_{9}H^{+}_{1}$	9	3	a11 3EI	0.20

811.61275	1	$C_{40}H_{78}N_{10}O_7H^+{}_1$	7	3	a10 3EI	-0.03
712.54415	1	$C_{35}H_{69}N_9O_6H^+{}_1$	6	3	a9 3EI	-0.29
613.47593	1	$C_{30}H_{60}N_8O_5H^+{}_1$	5	3	a8 3EI	-0.02
514.40734	1	$C_{25}H_{51}N_7O_4H^+{}_1$	4	3	a7 3EI	-0.37
415.33908	1	$C_{20}H_{42}N_6O_3H^+{}_1$	3	3	a6 3EI	-0.09
316.27076	1	$C_{15}H_{33}N_5O_2H^+{}_1$	2	3	a5 3EI	0.18
					Absolute Average	0.24
					Standard Deviation	0.37

Figure S 5: *a*-series of p(17Pox-4EI)OH

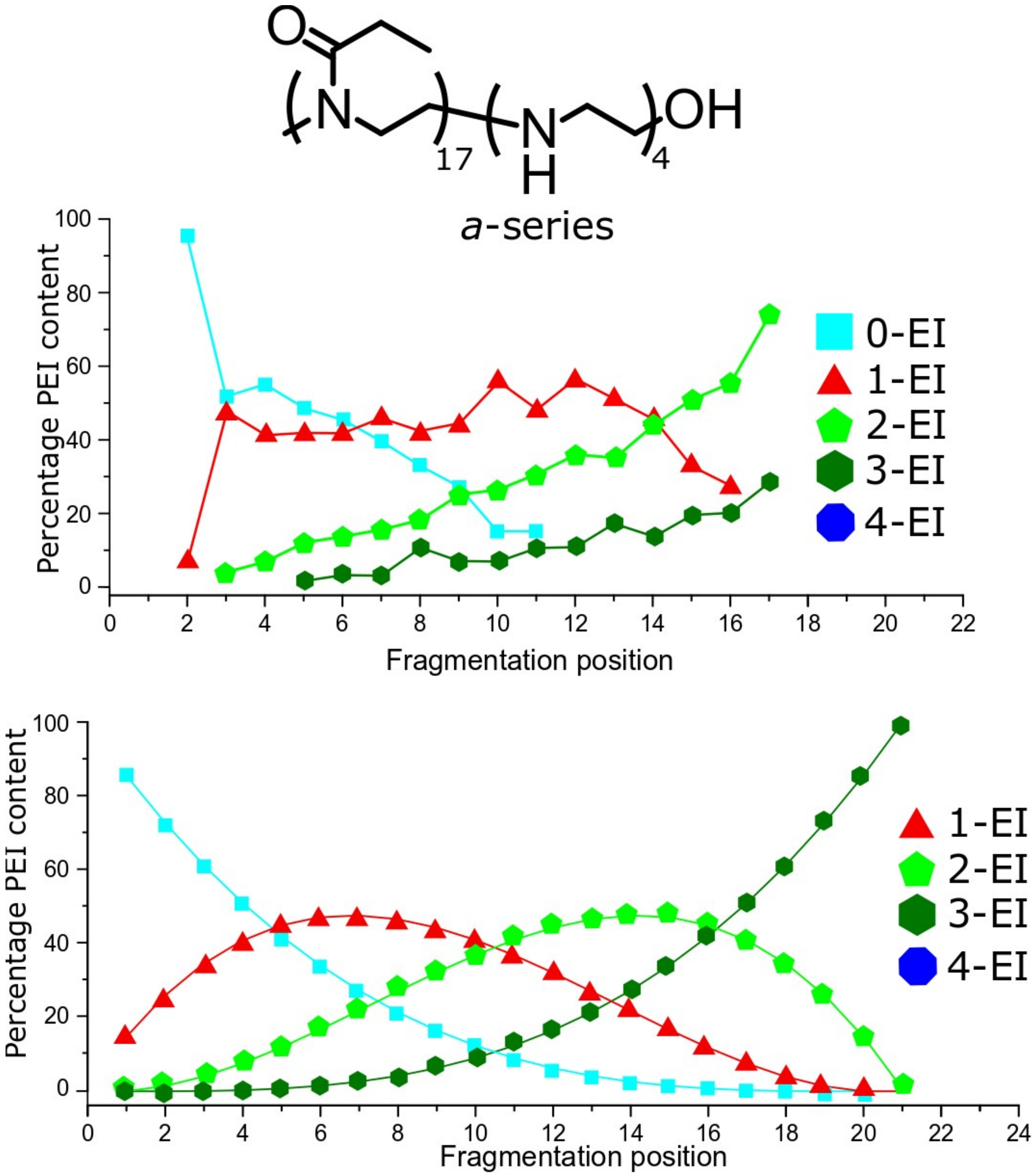

Section S7: Generation of combinations Python 3

```
# Python3 program to distinct combinations of inserted string

#Produce file

File_object = open(r"PermutationsofXY","a+")

def shouldSwap(string, start, curr):

  for i in range(start, curr):
    if stringi] == stringcurr]:
      return 0
  return 1


# Prints all distinct permutations

# in str0..n-1]

def findPermutations(string, index, n):
  if index >= n:
        #print(','.join(string))
    File_object.write(','.join(string) + "\n")
    #print(np.char.join(',',string))
    return


  for i in range(index, n):
        # Proceed further for stri] only if it doesn't match with any of the characters after strindex]

    check = shouldSwap(string, index, i)
    if check:
      stringindex], stringi] = stringi], stringindex]
      findPermutations(string, index + 1, n)
      stringindex], stringi] = stringi], stringindex]

# Driver code

if __name__ == "__main__":
  string = list("XY")
  n = len(string)
  findPermutations(string, 0, n)

File_object.close()


# This code is contributed by Rituraj Jain

#https://www.geeksforgeeks.org/distinct-permutations-string-set-2/ accessed 21st Jan 2020
```


[1] aC. Köster*, Vol. US 6188064 B1* (Ed.: U. S. Patent), Bruker Daltonik GmbH (DE), United States, **2001**; bC. A. Wootton, Y. P. Y. Lam, M. Willetts, M. A. van Agthoven, M. P. Barrow, P. J. Sadler, O. C. PB, *Analyst* **2017**, *142*, 2029-2037; cP. Kaur, P. B. O'Connor, *J. Am. Soc. Mass. Spectrom.* **2006**, *17*, 459-468.

[2] H. Glasner, C. Riml, R. Micura, K. Breuker, *Nucleic Acids Res.* **2017**, *45*, 8014-8025.

[3] B. R. Heap, *The Computer Journal* **1963**, *6*, 293-294.
